# Retino-protective effect of *Bucida buceras* against oxidative stress induced by H_2_O_2_ in human retinal pigment epithelial cells line

**DOI:** 10.1186/s12906-015-0765-6

**Published:** 2015-07-29

**Authors:** Simon Bernard Iloki-Assanga, Lidianys María Lewis-Luján, Daniela Fernández-Angulo, Armida Andrea Gil-Salido, Claudia Lizeth Lara-Espinoza, José Luis Rubio-Pino

**Affiliations:** Rubio Pharma y Asociados S. A de C. V., Laboratorio de Investigaciones en Bioactivos y Alimentos Funcionales (LIBAF), Blvd. García Morales Km. 6.5 # 330. El Llano. Hermosillo, Sonora, C.P. 83210 Mexico; Universidad de Sonora (UNISON) Hermosillo, Blvd. Luis Encinas y Rosales S/N Col. Centro, Hermosillo, Sonora C.P. 83210 Mexico

**Keywords:** *Bucida buceras* L, Retinal pigment epithelial cells, Oxidative stress, Hydrogen peroxide, Cellular redox status, Free radicals

## Abstract

**Background:**

Reactive Oxygen Species (ROS) impair the physiological functions of Retinal Pigment Epithelial (RPE) cells, which are known as one major cause of age-related macular degeneration and retinopathy diseases. The purpose of this study is to explore the cytoprotective effects of the antioxidant *Bucida buceras* extract in co-treatment with hydrogen peroxide (H_2_O_2_) delivery as a single addition or with continuous generation using glucose oxidase (GOx) in ARPE-19 cell cultures. The mechanism of *Bucida buceras* extract is believed to be associated with their antioxidant capacity to protect cells against oxidative stress.

**Methods:**

A comparative oxidative stress H_2_O_2_-induced was performed by addition and enzymatic generation using glucose oxidase on human retinal pigment epithelial cells line. H_2_O_2_-induced injury was measured by toxic effects (cell death and apoptotic pathway) and intracellular redox status: glutathione (GSH), antioxidant enzymes (catalase and glutathione peroxidase) and reducing power (FRAP). The retino-protective effect of co-treatment with *Bucida buceras* extract on H_2_O_2_-induced human RPE cell injury was investigated by cell death (MTT assay) and oxidative stress biomarkers (H_2_O_2,_ GSH, CAT, GPx and FRAP).

**Results:**

*Bucida buceras* L. extract is believed to be associated with the ability to prevent cellular oxidative stress. When added as a pulse, H_2_O_2_ is rapidly depleted and the cytotoxicity analyses show that cells can tolerate short exposure to high peroxide doses delivered as a pulse but are susceptible to lower chronic doses. Co-treatment with *Bucida buceras* was able to protect the cells against H_2_O_2_-induced injury. In addition to preventing cell death treatment with antioxidant plant could also reverse the significant decrease in GSH level, catalase activity and reducing power caused by H_2_O_2_.

**Conclusion:**

These findings suggest that *Bucida buceras* could protect RPE against ocular pathogenesis associated with oxidative stress induced by H_2_O_2_-delivered by addition and enzymatic generation.

## Background

Oxidative damage is involved in the pathogenesis of a variety of chronic degenerative and neurodegenerative diseases. Increasing evidence indicates that oxidative stress plays a major role in ocular pathologies including cataract, age-related macular degeneration (ARMD), glaucoma, and diabetic retinopathy (DR). Under normal physiological states, ocular tissues possess several intrinsic antioxidant enzymes to cope with oxidative stress formed as a consequence of normal metabolism. During ocular injuries, overproduction of reactive oxygen species (ROS) and free radicals overwhelms the intrinsic antioxidant mechanisms resulting in oxidative stress and ultimately development of a pathological condition [1–4].

There is a clear difference between ROS required for basic cellular mechanisms like cellular signaling and excessive ROS production that might cause oxidative stress and contribute to the pathogenesis of major diseases, including diabetes, neuro-degeneration and cancer [5]. Among the various ROS, hydrogen peroxide (H_2_O_2_) is perhaps the most ubiquitous of these species, which is found at measurable levels in all animal tissues. H_2_O_2_ is most stable and can reach molecular targets distant from its site of generation. Because H_2_O_2_ is a small, uncharged molecule, it easily crosses cell membranes and localizes in multiple subcellular compartment [6].

The effects of H_2_O_2_ are concentration dependent and range from physiological signaling such as cell proliferation, migration, survival, differentiation, and gene expression [7–10] to overt cell death [11, 12]. At nanomolar levels, H_2_O_2_ is a stimulant of cell growth and proliferation, whereas micromolar levels cause transient growth arrest and induce protective adaptive alterations in gene expression [13]. At millimolar levels, and above, H_2_O_2_ is clearly a toxic oxidant species, causing a frank oxidative stress. The different sensitivities of the cells to H_2_O_2_ are due to cell type, the species, and the differential antioxidant defense mechanisms to counteract the damaging effects of H_2_O_2_ concentrations.

Hydrogen peroxide treatment of cultured cells is a commonly used model to test oxidative stress susceptibility or antioxidant efficiency in cell types that are at high risk for oxidative damage in vivo, such as cells of the retinal pigment epithelium (RPE). Although the retina is a complex multilayered structure, it can be functionally divide in two parts: the neuronal retina, composed by photoreceptors (cones and rods) and their neuronal connections, is responsible for photo transduction process; the RPE and its basal lamina known as Bruch’s membrane maintain the integrity between retina and choroid. The RPE is composed of a polarized monolayer of pigmented hexagonal cells (melanin), and its integrity is essential for vision. Melanin in the RPE can act against ROS and protect the neural retina. Although the more popular mammalian RPE cell lines (e.g. ARPE19, D407, RPE-J) do not readily demonstrate melanogenesis, there have been numerous reports of repigmentation in ARPE19 and adult primary RPE cells [14–16].

The RPE is located adjacent to the outer retina, where it performs functions that are essential for the photoreceptor survival. Its main functions include nutrient, ion, and water transport; uptake of circulating vitamin A, its storage as an ester, its conversion to retinol, and then its transference to the photoreceptors; elimination of waste material accumulated by photoreceptors, diurnal phagocytosis and digestion of photoreceptor outer segment tips, light absorption, protection against photo-oxidation, and secretion of essential factors for maintaining the structural integrity of the retina [2, 3, 17, 18].

The retina is a part of the central nervous system (CNS), perceiving and processing visual information. But retinal photoreceptors are highly susceptible to oxidation process because they are exposed to a range of light intensities [19]. The RPE is at high risk of oxidative stress because it resides in an environment of high oxygen tension and is exposed to phototoxic blue light [20]. Among the reactive oxygen species to which the cells are exposed is hydrogen peroxide. As in most cells, H_2_O_2_ is generated during normal oxygen metabolism in mitochondria. In the RPE, H_2_O_2_ is also produced during daily phagocytosis of shed photoreceptor outer segments and is generated as a consequence of light irradiation of the pigment melanin. The retinal-pigmented epithelium is armed with a robust antioxidant system and may contain cellular defense mechanisms against ROS elevation. Glutathione and its related enzymes are part of this antioxidant defense. Moreover increased macular pigment density by lutein and zeaxanthin may reduce the exposure to blue light and subsequently reduce the photo-mediated production of reactive oxygen species [1, 2, 21, 22].

Although H_2_O_2_ addition to cell cultures is a common model of stress induction, its concentration in the medium over the period of cell treatment is usually not determined or controlled. The kinetics of H_2_O_2_ decomposition over short time frames has been examined in cultures of several monolayer cell types including the RPE [22, 23]. The major focus of this investigation is to evaluate the protective effect of *Bucida buceras* against H_2_O_2_-delivered by two methods: as a single addition pulse or by continuous enzymatic generation on retinal pigment epithelial cells (ARPE-19) using cells death and oxidative stress indicators.

*Bucida buceras* L. (Combretaceae) is widely planted for shade and ornamental use in America. Very few studies have been done with *Bucida buceras*, but it contains functional components such as flavonoids and terpenes responsible of potent cytotoxicity activity against human tumor cell lines [24, 25]. Some authors have reported that extracts of *B. buceras* have antibacterial and antifungal activities (Adonizio et al., 2006; [26–28]). Our previous phytochemical screening of *Bucida buceras* give evidence of the presence of carotenes, triterpenes/steroids, lactonic groups, phenols/tannins, amines or amino acids, flavonoids/anthocyanins, saponins and reducing compounds in different extracts of *B. buceras.* These phytochemical compounds may be associated with the strong antioxidant potential found in this plant and it will contribute to protect against ocular pathogenesis associated with oxidative stress. The effect of *Bucida buceras*, in retinal health has not been studied.

## Methods

### Material

Hydrogen peroxide, catalase (from bovine liver), glucose oxidase (from Aspergillus niger), 3-(4,5—dimethylthiazo-2-yl)-2,5-diphenyl tetrazolium bromide (MTT), Dulbecco’s modified Eagle’s medium (DMEM), fetal bovine serum (FBS), pen`icillin, streptomycin, L-glutamine, trypsin-EDTA, iron (II) ammonium sulfate hexahydrate, xylenol orange disodium salt (3,3¨-bis(N,N-di(carboxymethyl)-aminomethyl)-o-cresulfone-phatein), phosphate-buffered saline (PBS) 5,5′-dithiobis(2-nitrobenzoic acid (DTNB), 2,2 difenil-1-picrilhidrazil (DPPH), and 2,4,6-tripyridyl-s-triazine (TPTZ), were purchased from Sigma (St. Louis, MO, USA). RIPA buffer from Thermo Scientific, caspase-3-substrate (Ac-DEVD-pNA) from Santa Cruz Biotechnology (Santa Cruz, CA, USA) and caspase-3 inhibitor I (DEVD-CH0) from Calbiochem (Merck, Millipore).

### Cell cultures

The hRPE cell line, ARPE-19 (ATCC, 2302) was kindly gifted from Dr. Horacio Rilo (Arizona, University, USA) and was used during all experiments. The cells were propagated in T 25 cm^2^ culture flacks using twice-weekly feedings of DMEM containing 10 % fetal bovine serum and antibiotics (penicillin 100 U/ml and streptomycin 0.1 mg/ml) at 37 °C in a humidified 5 % CO_2_ atmosphere. Attached and floating cells in the experimental dishes were collected after trypsinization with 1x trypsin-EDTA, centrifuged, re-suspended in fresh medium and counted using trypan blue exclusion assay.

### Plant collection and extract preparation

*Bucida buceras* L. was collected in April-May 2013 from Sonora, Mexico. The plant material was botanically identified by Dr. Jose Cosme Guerrero, department of Agriculture and Livestock, Sonora University (UNISON). A voucher specimen (RP-2014-10) was deposited at the Herbarium of UNISON.

The leaves were collected fresh and shade dried to obtain 500 g dry sample, then was later coarsely powdered in an electric chipper shredder (Chicago electric power tools) and used for solvent extraction. For sample preparation, 500 g of dried sample were extracted twice (1000 ml for each with absolute ethanol at 25 °C for 1 week and concentrated using a rotary evaporator (Buchi R-210, Switzerland) at 37 °C and preserved at 4 °C. Dried sample of ethanol extract (200 mg) was dissolved 1 ml of dimethyl sulfoxide as a stock solution and stored at −20 °C.

### Determination of cell viability via trypan blue method

Viability and cell numbers were determined by staining cells with trypan blue dye. Cells were harvested, collected and re-suspended in 3 ml medium. Then the cells were stained with a final concentration of 0.04 % for 5 min at room temperature. Stained and unstained cells were counted using a hematocytometer in optic microscope. Unstained cells represent the viable cells, which were not permeable to trypan blue dye.

### H_2_O_2_ exposure on ARPE-19 cells

In H_2_O_2_ treatment experiments, the cells were plated at 5×10^4^ cell/well in 96-well plates to adhere overnight before exposure to oxidant. Two treatment protocols were used: pulse delivery of a range of micro-molar and milli-molar concentrations of H_2_O_2_ and addition of glucose oxidase to initiate continuous enzymatic generation of the oxidant.

For pulse delivery, the culture medium was first removed and cells were fed with DMEN in presence or absence of serum (10 %) and exposed to different ranges of concentrations of H_2_O_2_, freshly prepared in medium: treatment 1-(12.5, 25, 50, 100, 200, 400, 800, 1200, 1600 μM) during 24 h with intervals of up to 30 min during the first 2 h and treatment-2 (0.01, 0.05, 0.1, 0.5, 1, 5, 10, 25 mM) in 30, 60 and 120 min. Control cells were cultured in H_2_O_2_-free medium and H_2_O_2_ was also added to medium in culture wells lacking cells to determine H_2_O_2_ depletion in the absence of culture monolayers.

For continuous enzymatic generation of H_2_O_2_, the cultures were first refed with fresh DMEM containing 10 % FBS (D-10). Glucose oxidase (GOx) was then added to the medium to initiate the generation of H_2_O_2_ by oxidation of the glucose contained in DMEM (4.5 mg/ml D-glucose). Stock solutions of GOx were prepared by solubilizing the enzyme in 50 mM sodium acetate buffer, pH 5.1, at a concentration of 10 KU/ml and storing aliquots at −20 °C. Just before use, stock solutions were thawed, diluted, and added to the culture medium to produce final concentrations of 3–100 mU/ml. GOx was added as well to medium in culture wells lacking cells to determine H_2_O_2_ depletion in the absence of culture monolayers.

After addition of H_2_O_2_ (pulse delivery) or GOx (to initiate continuous H_2_O_2_ generation), aliquots of culture medium were retrieved at intervals to determine H_2_O_2_ levels, and cells were harvested after 24 h (treatment-1 and GOx) or 2 h (treatment-2) to perform cytotoxicity assays by the methods described below.

### Hydrogen peroxide determination

Hydrogen peroxide concentration in culture medium was determined by a modified ferrous oxidation-xylenol orange (FOX) assay reported by Gil et al. [29]. Complete FOX reagent consisted in a mix of two solutions: contained 25 mM ammonium ferrous sulfate in 3.5 M H_2_SO_4_ (solution A) and 0.125 mM xylenol orange disodium salt (solution B). Just before use 1 volume of solution A was added to 100 volumes of solution B to produce complete FOX reagent. For the assay, an aliquot of medium retrieved from cultures (50 μl DMEM or D10 in the presence and absence of ARPE-19 cells) were mixed with 500 μl of complete FOX reagent.

The oxidation of Fe^2+^ in the presence of hydroperoxides forms Fe^3+^, which reacts with xylenol orange to produce a chromosphere. Samples were incubated for 30 min at room temperature. Absorbance of the supernatant was read spectrophotometrically at 560 nm against PBS as background control. 50 μl of known concentrations of H_2_O_2_ (3.125 to 100 μM) were used as standard calibration curve and a best-fit line for the data was plotted using linear regression. Experimental H_2_O_2_ concentrations were then extrapolated substituting the unknown values into the equation derived for the standard curve.

### Assay of mitochondrial viability (MTT assay)

In the MTT assay, cell respiration, and thus cell viability, was assessed based on the mitochondrial-dependent reduction of MTT to form the colored product (purple) reported by Mossman [30]. Cytotoxicity was determined in different times after treatment of ARPE-19 cultures with H_2_O_2_, GOx or co-treated with the indicated concentrations of *Bucida buceras* ethanol extract. Afterward the medium was replaced by MTT (0.5 mg/ml in medium), and the conversion of MTT into an insoluble formazan crystals for 4 h at 37 °C were solubilized in acidified isopropanol. The absorbance was monitored at 570 nm and 655 nm in a microplate reader (Thermo Scientific Multiskan Spectrum). Cell viability was expressed as a percentage of the control value (untreated cells), which was set to 100 %. Control cells were cultured in medium supplemented with 0.1 % dimethyl sulfoxide, which was used as the solvent for the extract.

### Preparation of cell lysates from ARPE-19 cells and protein quantification

One million cells were plated in 12-well plates and allowed to adhere overnight before hydrogen peroxide-stress. Cultures were treated with H_2_O_2_ (800, 1000, 1600 μM) in addition or GOx (12.5, 25, 50 mU/ml) alone or cells treated with H_2_O_2_ followed by co-treatment with *Bucida buceras* extract (1600 μg/ml) for 24 h. Control cells were cultured in medium in absence of H_2_O_2_. After incubation time the medium was carefully removed and washed twice with cold PBS buffer, trypsinized and centrifuged at 1500 rpm for 5 min. For making whole cell lysates, the treated and untreated cells were lysed in phosphate-buffered saline (PBS; 136.75 mM NaCl, 2.68 mM KCl, 1.47 mM KH_2_PO_4_, 8.10 mM Na_2_HPO_4_, pH 7.4) by three freeze-thaw cycles (−77 °C/25 °C) and then disrupted by sonication. The homogenate was centrifuged at 14,000 g at 4 °C for 15 min. Protein concentration determination was performed by the Bradford method using bovine serum albumin as the standard at 595 nm [31].

### Measurement of apoptosis/caspase-3 assay

After treatment the medium was aspirated and cells were washed once in cold PBS and afterward counted using the trypan blue exclusion method. An amount of 1x10^6^ cell/ml was transferred to lysis RIPA buffer. The activity of caspase-3-like protease in the lysate was measured using colorimetric caspase-3 assay kit according to the reported by Bai et al. [32]. In brief, cytosolic protein (100 μg) was mixed with caspase-3-specific substrate acetyl-Asp-Glu-Val-Asp-p-nitronilide (final concentration, 200 μM) and incubated at 37 °C for 90 min. The absorbance was read at 405 nm. Apoptotic cell lysates containing active caspase-3 produce a considerable activity compared to non-apoptotic cell lysates.

To confirm that substrate cleavage was due to caspase-3 activity, extracts were incubated in the presence of caspase-3-specific inhibitor acetyl-DEVD-CHO (final concentration, 20 μM) at 37 °C, before the addition of substrate. The value (in arbitrary absorbance units) of the absorbance signal of the inhibited sample was subtracted from the no inhibited sample.

### Antioxidant activities

#### Hydrogen peroxide (H_2_O_2_) scavenging activity assay

Hydrogen peroxide scavenging activity of *Bucida buceras* ethanol extract was measured by the FOX assay [29]. H_2_O_2_ (100 μL of 1600 μM or GOx 50 mU/ml) and 100 μL of various concentrations (400, 800 and 1600 μg/ml) of the extract were mixed. After 1 h, 50 μl were mixed with 500 μl of complete FOX reactive and incubated for 30 min at room temperature.

### Total antioxidant capacity

Total antioxidant capacity in the lysates cells and extract were assayed using the FRAP assay of Benzie and Strain [33] with some modifications according to Iloki et al. [34]. The FRAP reactive was prepared in acetate buffer (pH 3.6), 10 mmol 2,4,6-tripyridyl-s-triazine (TPTZ) solution in 40 mmol HCL and 20 mmol iron (III) chloride solution in proportions of 10:1:1 (v/v), respectively. The FRAP reactive was prepared fresh on a daily basis and warmed to 37 °C in water batch prior to use. 5 μl of sample diluted with 15 μl of PBS were added to 150 μL of FRAP reagent. The absorbance of the mixture was measured using a microplate spectrophotometer reader Thermo Scientific Multiskan Spectrum at 595 nm after 5 min. The standard curve was prepared by ascorbic acid (AA) solution, and the results were expressed as μMAA/mg extract.

### DPPH-scavenging capacity

*Bucida buceras* ethanol extract was evaluated for its activity to scavenge the stable DPPH radical (0.1 mM in ethanol) according to a previously described method [35] with some modifications according to Lewis et al. [36]. The affinity of the test material to quench the DPPH free radical was evaluated according to the equation scavenging % = (A_c_-A_s_)/A_c_*100 %. A_s_ and A_c_ are the absorbance at 517 nm of the reaction mixture with sample and control respectively.

### Redox homeostasis

#### Cellular thiol quantification

Total intracellular thiol levels were quantified spectrophotometrically using 5,5′-dithiobis (2-nitrobenzoic acid (DTNB)), as described previously by Gil et al. [29] with glutathione reduced (GSH) as standard. Briefly, this method is based on the reaction of the glutathione present in the lysates with DTNB to generate oxidized GSH (GSSG) and 2-nitro-5-thiobenzoic acid, a yellow compound that absorbs at 412 nm.

### Activity of antioxidant enzymes

Catalase (CAT) activity was measured by using a protocol adapted from [29]. Catalase activity was assayed adding 50 μl of sample to 450 μl of 50 mM phosphate buffer, pH 7.0, and 250 μl of 50 mM H_2_O_2_ in a final volume of 750 μL. Absorbance decrease was measured at 240 nm in a Cary spectrophotometer for 1 min and calculations were performed using an extinction coefficient of 0.043 mM^−1^ cm^−1^. Catalase activity is expressed as μmol of H_2_O_2_ /min*ml. All experiments were carried out using three biological replicates and three technical replicates for each sample.

### Assay of GSH-peroxidase activity

GSH (200 μM) and H_2_O_2_ (200 μM) were incubated at 37 °C for 1 h in PBS containing various lysates of ARPE-19. The volume of the enzymatic reaction was 120 μl. Residual peroxide was measured spectrophotometrically at 560 nm by a modified ferrous oxidation-xylenol orange (FOX) assay.

### Microscopic imaging

Samples from ARPE-19 cells treated or untreated with H_2_O_2_ and *Bucida buceras* were evaluated using an inverted microscope (Faga-Lab-Labomed, TCM 400) to detect structural changes. A series of structural images were collected with a bottom-mount digital camera for the analysis of treatments.

### Statistical analysis

Data is expressed as the mean ± standard deviation (SD). Significance between experimental groups was determined by GLM-ANOVA followed by post hoc Tuckey test using NCSS 2007 software. Values of p ≤ 0.05 were considered statistically significant. Figures were performed using Origin 8.0 software and the error bars represent the SD.

## Results

### Pulse delivery of H_2_O_2_

**Fig. 1:**
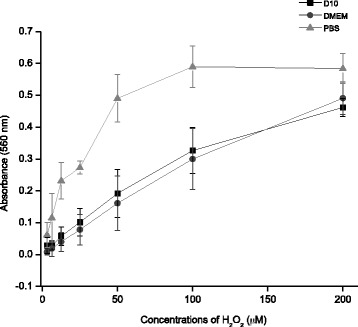
Calibration curves for H_2_O_2_ levels in the range 10–200 μM using ferrous-xylenol orange with 10 % FBS (D10; linear regression y = 0.0022x + 0.0149, R^2^ = 0.98616 and without FBS (DMEM linear egression: y = 0.0025x + 0.0157, R^2^ = 0.98643. Therefore H_2_O_2_determination was measured in PBS. Data are from three independent experiments and are the means ± SD of three culture wells per group in each experiment

Preliminary experiments were conducted to confirm that the presence of serum did not affect H_2_O_2_ measurement. As shown (Fig. 1), hydrogen peroxide determinations did not differ for a range of concentrations of H_2_O_2_ (10–200 μM) added to DMEM either lacking serum or containing 10 % FBS. However previous H_2_O_2_ concentrations in PBS are nearly at an order of magnitude higher than that in medium. It is not clear which component(s) of DMEM is/are responsible for the slow but measurable decomposition of H_2_O_2_ in the medium.

**Fig. 2:**
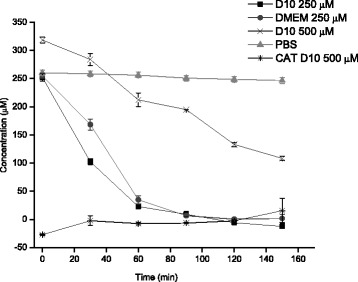
Degradation of 250 μM H_2_O_2_ added as single pulse to medium without serum (DMEM) and with 10 % FBS (D10) in the absence of ARPE-19 cells. Exponential regression of 250 μM H_2_O_2_ in D10 y = 245.54e^-0.029x^, R^2^ = 0.96447. Therefore H_2_O_2_ determination was measured in PBS. Addition of catalase 200 U/ml to the 500 μM H_2_O_2_ with serum confirms the specificity of the determinations for H_2_O_2_. Data are from three independent experiments and are the means ± SD of three culture wells per group in each experiment

Exogenously added of H_2_O_2_ has a short half-life as a result of its rapid degradation in culture medium. For monitoring the decay of the H_2_O_2_ bolus in our experiments and to know how long and to what extent H_2_O_2_ levels remain elevated after a bolus treatment, the kinetics of H_2_O_2_ degradation was determined in the presence and absence of cells. In the absence of cells, the initial concentration is not sustained in the medium (Fig. 2) and therefore the concentration of the agent and the time of exposure are not well controlled. As illustrated for an initial nominal concentration of 250 μM, the depletion rate is almost similar over 2 h at 37 °C in the absence and presence of serum. Although, in the presence of serum (D10) H_2_O_2_ concentration undergoes a more rapid exponential depletion, the half time of depletion is 30 min with 10 % FBS while in DMEM (without serum) it is 45 min. The rapid degradation of H_2_O_2_ in medium with serum dependent on its own serum antioxidant joined to its amino acids present in the medium. H_2_O_2_ concentration in PBS declines slowly with time.

The rapid elimination of H_2_O_2_ in culture medium is dependent of initial nominal concentrations. While 250 μM, was totally diminished in the medium in the first our; an increases in H_2_O_2_ concentration (500 μM) delay the rapid degradation but during the time was eliminated. Because of oxidation of ferrous to ferric ions in biological samples could also result from the interaction of ferrous ions with organic hydroperoxides, H_2_O_2_ specificity was confirmed by measuring concentrations of 500 μM H_2_O_2_ in D10 without and with catalase at a final concentration of 200 U/ml in the assay mix (Fig. 2). Catalase (CAT) broke down H_2_O_2_ into H_2_O and O_2_. Catalase is likely to be of particular relevance when cells are exposed to high H_2_O_2_ concentrations, on account of its essentially non-saturable first-order kinetics.

**Fig. 3:**
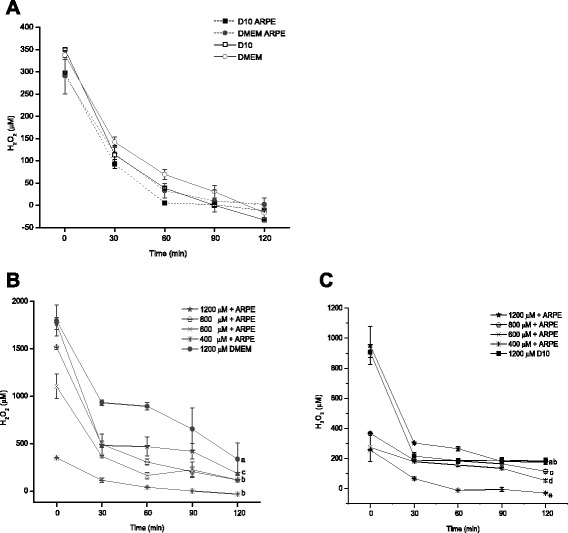
Dissipation of 400 μM nominal concentration of H_2_O_2_ (**a**), different nominal concentrations 400, 600, 800 and 1200 μM with serum-free medium (**b**) and with 10 % FBS (**c**) from the culture medium in presence o absence of ARPE-19 cells (50, 000 cell/well) with o without serum. Decay rate constants were k_d_ = −0.065, −0.041, −0.037, −0.026 min^−1^ for D10 + ARPE-19 cells, D10, DMEM + ARPE-19 cells and DMEM respectively. Data represent means of triplicate measurements of 3 independent experiments. 1200 μM of H_2_O_2_ in absence of ARPE-19 cells was determined in parallel culture

The relationship between H_2_O_2_ concentration and incubation time shows an exponential decline (Fig. 3). Exponential decay was more rapid in medium with serum in presence of ARPE-19-cells (k_d_ = −0.065 min^−1^), similar decay rate constants (k_d_) were found in medium with 10 % of serum and DMEM without serum in presence of ARPE-19 cells (−0.041 and −0.037 min^−1^ respectively) (Fig. 3a). The near perfect exponential decay (shown for 400 μM bolus) in Fig. 3a demonstrates that H_2_O_2_ degradation closely follows first-order kinetics with dependence on substrate concentration. Fitting of the measured concentrations to the function.

### [H_2_O_2_] = [H_2_O_2_]_ini_ x e-^kt^

Where [H_2_O_2_]_ini_ is the initial concentration and k (min-^1^) the first order rate constant. The rate constant is used to calculate elimination half-life (t_1/2_) and the time of 99 % H_2_O_2_ disappears from the supernatant (t).

- t_1/2_ = ln2/k, t_1/2_ = ln2/0.065, t_1/2_ = 0.693/0.065 t_1/2_ = 10.67 min
- t = ln(0.01)/-k; t = ln(0.01)/-0.065; t = 70.85 min

For ARPE-19 cells grown in DMEM with 10 % FBS using the specific elimination rate constant, k_d_ = −0.065 min^−1^ derived from the relationship between peroxide concentration and the time (Fig. 3), it can be estimated that the half-life elimination (t_1/2_) was 10.67 min and the elimination total time was 70.85 min. By this time the concentration of H_2_O_2_ in culture medium had diminished more than 10-fold.

ARPE-19 cells were exposed to a variety of H_2_O_2_ nominal concentrations (400–1200 μM), by delivering oxidant to culture as a single addition (pulse) with serum-free culture medium (Fig. 3b) and with 10 % of serum (Fig. 3c). The concentration-effect relationship shows that the response of cultured cells is probably determined by both the concentration of the agent and the time of exposure (Fig. 3).

A rapid exponential depletion of H_2_O_2_ from serum-free medium exposed to RPE culture was obtained (Fig. 3b), although elimination of H_2_O_2_ in ARPE-19 cell culture is much more rapid in the presence of serum (Fig. 3c). The concentration of H_2_O_2_ in the medium of ARPE-19 cell cultures with serum starts to decrease immediately after administration of the peroxide. At least for the first 30 min of incubation H_2_O_2_ disappears from the culture. By 2 h the concentration of H_2_O_2_ in culture medium had diminished dramatically, but residual high H_2_O_2_ concentrations remain in serum-free media.

An initial exponential decline of the H_2_O_2_ concentration in the culture medium was observed with H_2_O_2_ concentrations between 100 and 1600 μM. In general, concentrations ≤100 μM H_2_O_2_ were completely eliminated during the first 30 min after administration. At initial H_2_O_2_ concentrations of ≥ 200 μM after the initial rapid phase, the elimination became slower and the concentration of H_2_O_2_ leveled off. Regardless of the kinetics, from the pragmatic point of view, the significant observation is that H_2_O_2_ is not sustained at the added concentration when the agent is delivered as a pulse.

### Influence of cell concentration on the elimination H_2_O_2_ concentrations

**Fig. 4:**
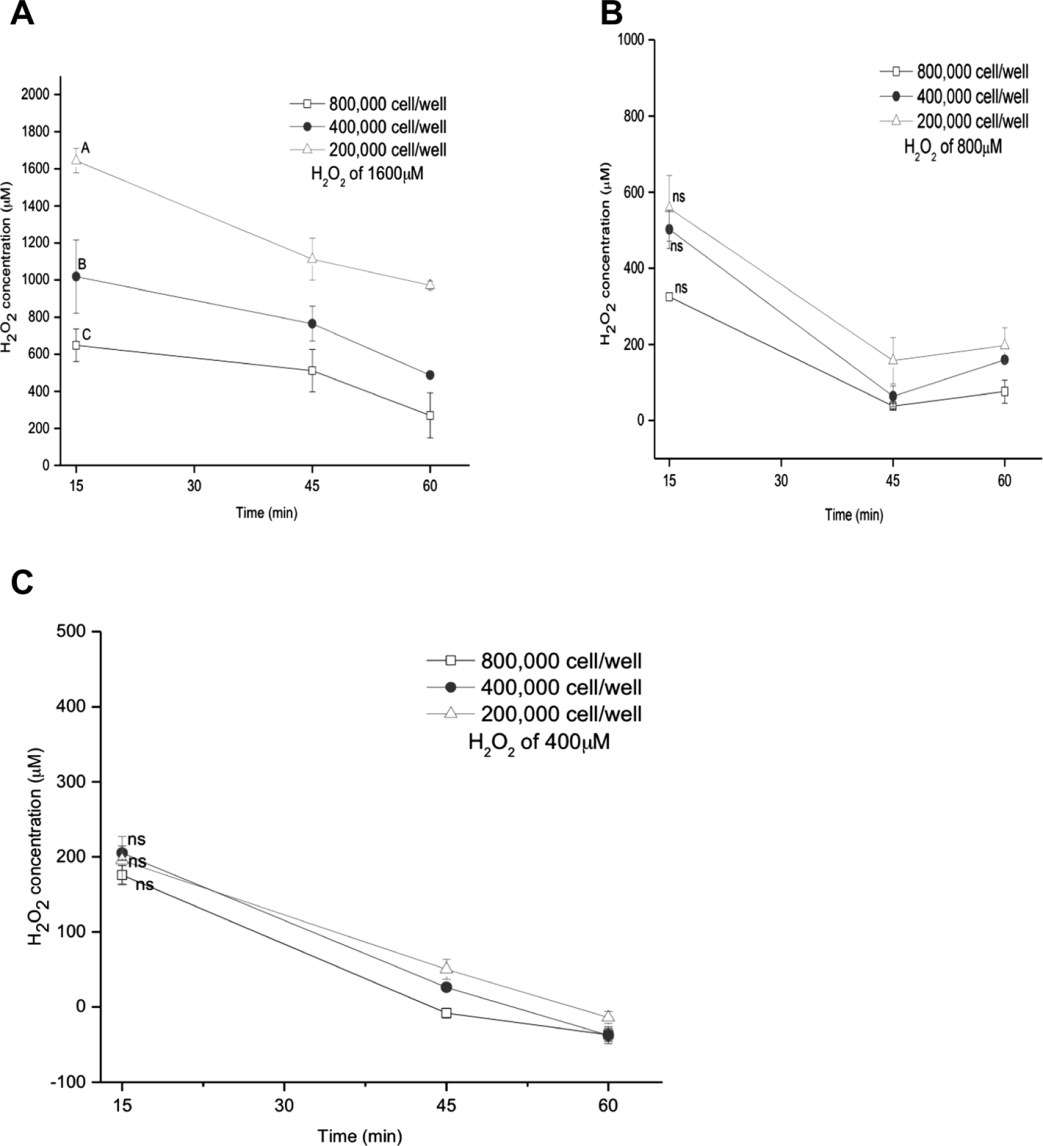
Influence of cells number on the elimination of hydrogen peroxide in ARPE-19 cells. Cells were incubated with various concentrations of H_2_O_2_ 1600 μM (**a**), 800 μM (**b**) and 400 μM (**c**) for the indicated times. Data are means ± SD of three independent experiments

ARPE-19 cultures with different cell number (2, 4, 8 ×10^5^ cells/well) were exposed to various nominal H_2_O_2_ concentrations (400, 800, 1600 μM) in 1 ml /well to evaluate the elimination of H_2_O_2_ from culture medium and the cytotoxic action. Figure 4 shows that increasing the cell concentration accelerate the peroxide depletion. Raising the cell concentration by increasing the cell number resulted in a shift of the nominal concentration-effect curves for the elimination of H_2_O_2_ toward higher concentrations (Fig. 4a, b). The elimination of H_2_O_2_ changes with variation of cell number, although this effect is almost independent of the cell number when the dose of H_2_O_2_ is low (Fig. 4c).

### Cytotoxicity in addition of H_2_O_2_

**Fig. 5:**
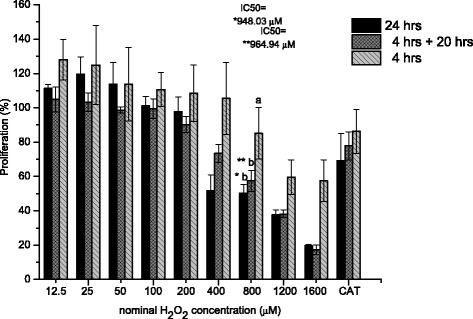
Cytotoxic action of hydrogen peroxide in ARPE-19 cells. A range of concentrations of H_2_O_2_ from 12.5 to 1600 μM was added to serum-containing DMEM. Cytotoxicity was estimated 24 h after addition by the MTT assay in cultures in which the medium was not replaced (solid bars) or was replaced at 4 h with fresh H_2_O_2_-free DMEM (hatched bars) or was measured at 4 h (gray-hatched bars). Addition of catalase 200 U/ml to the 1600 μM H_2_O_2_ as CAT control cells. * IC_50_ in 24 h ** IC_50_ 4 h with replace (4 h + 20 h). Data expressed as a percentage of the untreated control, are from three independent experiments and are the means ± SD

It is well established that H_2_O_2_ added to the medium of ARPE-19 cells as a single pulse produces an oxidant-dependent cytotoxicity that can be detected by MTT assay 24 h after treatment (Fig. 5). Because H_2_O_2_ is largely depleted from culture medium within 2 h (Fig. 3), one would expect similar cytotoxicity whether the medium was replaced with fresh medium at 4 h after oxidant addition or remained unchanged until the time of assay.

The figure reveals that H_2_O_2_ concentrations ≤100 μM are not cytotoxic at 24 h when H_2_O_2_ is delivered as a pulse, since the oxidant that is depleted fairly rapidly in these concentrations in the culture medium. Additionally ARPE-19 cell is not very sensitive to oxidative stress. Although 200 μM had little effect, but cytotoxicity was substantial at higher concentrations. The minimal concentration necessary to elicit 50 % of the cytotoxic effect (IC_50_) was calculated to be 948 μM at 24 h similar to 964 μM whether the medium was replaced with fresh medium at 4 h after oxidant addition. As shown, similar outcomes were in fact obtained (Fig. 5). The manifestation of the incipient cytotoxicity of H_2_O_2_ at 4 h means that cytotoxicity of H_2_O_2_ takes several hours longer than the exposure to H_2_O_2_

In the case of H_2_O_2_, incubation time is not equivalent to exposure time when oxidant is delivery as single addition. The clearance measurements reveal that H_2_O_2_ that is rapidly eliminated obviously the cell death is not immediate and the window between initial exposure and cell death is relevant because it is in this interval that antioxidants could function to increase the fraction of surviving cells.

**Fig. 6:**
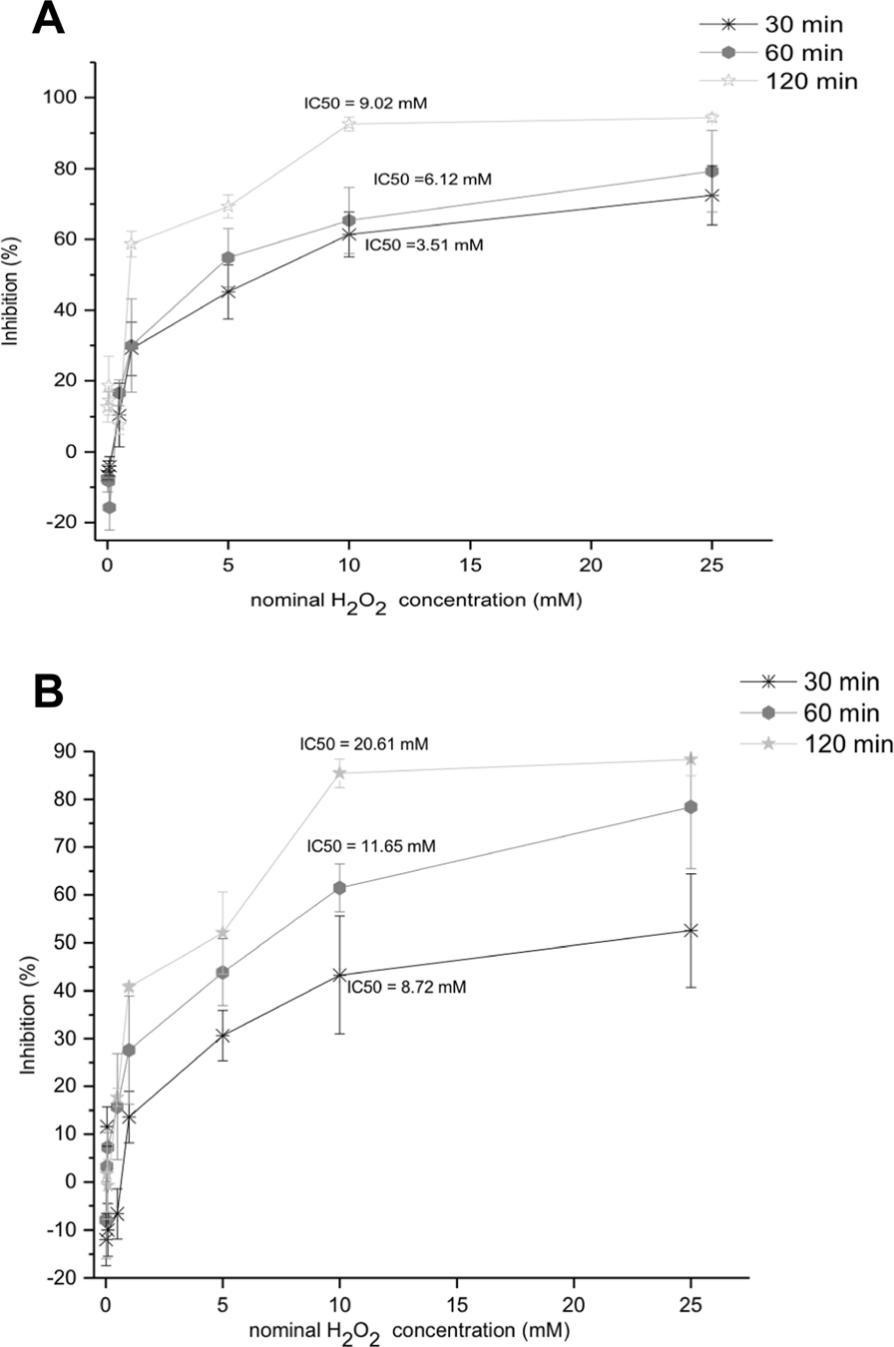
Influence of incubation time on the cytotoxic action of hydrogen peroxide in ARPE-19 cells. 50,000 cells were incubated for the indicated time (30, 60 and 120 min) with various concentrations of the peroxide (0.01, 0.05, 0.1, 0.5, 1, 5, 10, 25 mM) in free serum culture medium (**a)** and with serum (**b**). Data are means of at least three independent experiments

Additionally the ARPE-19 cells were exposed to a short exposure time (30, 60 and120 min) with H_2_O_2_ with or without serum to find the cytotoxic effect (Fig. 6). The amount of H_2_O_2_ that has to be added to ARPE-19 cell cultures to produce 50 % cytotoxicity increased about 10-fold by shortening the incubation time. Exposure to H_2_O_2_ concentrations ≤ 0.5 mM showed little effect on both culture cells, however, 5 mM H_2_O_2_ exposure by 60 and 120 min resulted in a significant loss of cell viability, with a 50 % reduction in serum-free culture compared to 10 mM for the ARPE-19 cells containing serum in the same short periods. Increasing exposure to 25 mM resulted in an 80 % loss of viability in ARPE-19 cells in both cultures types at 120 min. Short-term exposure to high H_2_O_2_ levels induced marked toxicity, suggesting that under these experimental conditions necrosis was the predominant mode of cell death.

The IC_50_ value revealed that the cytotoxic potency in serum-free culture medium was increased nearly two-fold over the ARPE-19 culture grown in 10 % FBS (Fig. 6). This high sensitivity to oxidation was attributed to serum starvation-induced apoptosis owing to the lack of serum antioxidants. The duration of incubation with H_2_O_2_ is important for the determination of H_2_O_2_ concentration required to induce cellular death.

### Continuous enzymatic generation of H_2_O_2_

Because H_2_O_2_ is labile in culture medium, sustained exposure to a given concentration is difficult to achieve when the agent is delivered in a single pulse. An alternative method for sustained treatment of cultures is to continuously generate the product from glucose medium using GOx. Glucose oxidase catalyzes the direct two-electron reduction of oxygen to H_2_O_2_, using reducing equivalents from the oxidation of glucose. This approach has been used for short-term treatment of cultured cells including, ARPE-19 cells [30].

**Fig. 7:**
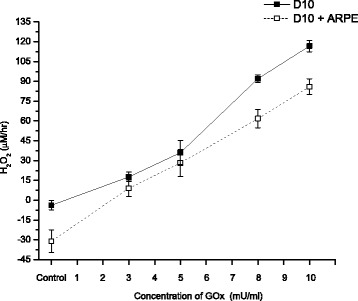
H_2_O_2_ concentration in culture medium generated from D-glucose after 1 h post addition of 0–10 mU/ml glucose oxidase (GOx). GOx was added to the medium of culture well without or with confluent cultures of ARPE-19 cells to illustrate the initial linear accumulation of H_2_O_2_ as a function of enzyme concentration. Data are from three independent experiments and are the means ± SD of three culture wells per group in each experiment

The addition of GOx to culture medium (DMEM) produces a linear rate of accumulation of H_2_O_2_ as a function of enzyme concentration during the first hour of incubation in both, with the absence and the presence of ARPE-19 cells (Fig. 7), although the rate of accumulation in the absence of cells is approximately twice as great as when cells are present. The generation of H_2_O_2_ by GOx can be assuming pseudo-zero order kinetics (i.e., assuming an unlimited supply of Glc and O_2_).

**Fig. 8:**
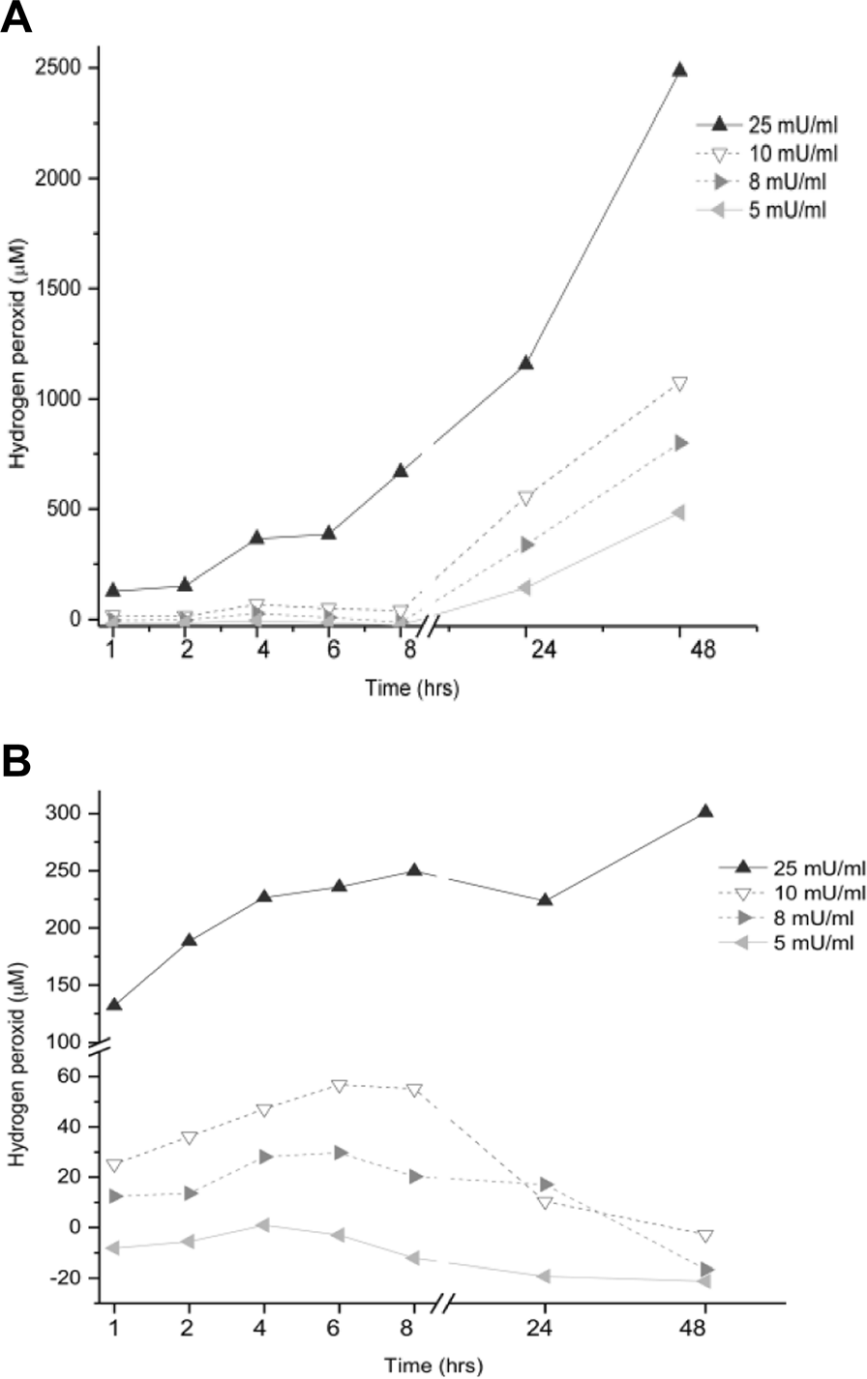
H_2_O_2_ concentration in culture medium generated from D-glucose after addition of 5, 8, 10 and 25-mU/ml glucose oxidase (GOx) during 48 h. GOx was added to the medium of culture wells (**a**) without or (**b**) with confluent cultures of ARPE-19 cells. Because higher medium concentrations are achieved in the absence of cells, different y-axis scales are used. Data are from three independent experiments and are the means ± SD of three culture wells per group in each experiment

In the absence of cells, H_2_O_2_ concentration continues to increase over 48 h at a higher rate than during the first hours, perhaps owing to the partial H_2_O_2_ depletion by medium that overcame by hydrogen peroxide accumulation for glucose oxidase (Fig. 8a). In the presence of cells the concentration of H_2_O_2_ in the culture medium over time after GOx addition is strikingly different in the absence of cells, and the pattern differs with enzyme concentration (Fig. 8b).

With a lower amount of GOx (5 mU/ml) H_2_O_2_ concentration in the medium is nearly stationary in the presence of cells during 48 h. The production rates and decomposition of H_2_O_2_ are approximately equal. At middle GOx amounts (8 or 10 mU/ml), H_2_O_2_ concentration exhibits a complex dynamic. The concentration continues to rise after the first hour of enzyme addition, peaks at approximately 6 h, and then decreases thereafter. Using 10 mU/ml GOx delivery to the ARPE-19 cultures a maximum concentration of 60 μM is observed at 6 h and this declined to 0 μM by 48 h. With higher concentration (25 mU/ml) the rate of H_2_O_2_ production continues to increase over 48 h, reaching a maximum value of 280 μM much lower than the seen in the absence of cells (Fig. 8b). These changes in medium content of H_2_O_2_ are related to the cytotoxic response of ARPE-19 cells.

### Cytotoxicity in enzymatic generation of H_2_O_2_

**Fig. 9:**
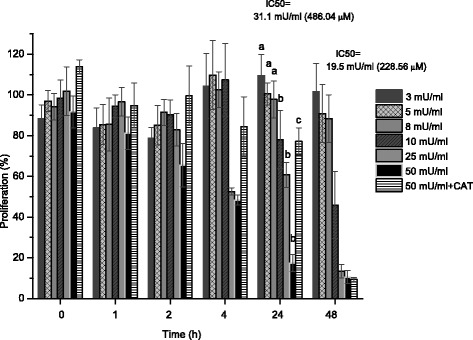
Influence of glucose oxidase concentrations on the cytotoxic action of hydrogen peroxide in confluent cultures of ARPE-19 cells. The viability was determined as a percentage of the untreated controls using the MTT assay for the indicated times. IC_50_ values have 31.1 mU/ml and 19.5 mU/ml at 24 h y 48 h respectively. Data are from at least three independent experiments and are the means ± SD of three culture wells per group in each experiment

As for delivery of H_2_O_2_ in a single pulse (Fig. 5), GOx addition to culture medium also produces a dose-dependent cytotoxic response in confluent ARPE-19 cultures quantified by the MTT assay at different times (Fig. 9). Low amounts of added enzyme (3 or 5 mU/ml) had little effect, but cytotoxicity was substantial at higher concentrations. This observation is noteworthy because the peak concentrations of H_2_O_2_, achieved at 6 h after addition of middle concentrations enzyme (Fig. 8b), were relatively low compared to concentrations that produced cytotoxicity when H_2_O_2_ was added in a single pulse (Fig. 5).

Using 19.5 mU/ml GOx, the concentration of 228.6 μM of H_2_O_2_ produced by this amount of enzyme reduced MTT in 50 % at 48 h, while 31.1 mU/ml achieved 486.04 μM of H_2_O_2_ with almost 50 % of inhibition at 24 h (Fig. 9), yet 900–1000 μM H_2_O_2_ was required to produce comparable MTT reductions when the oxidant was delivered as a pulse (Fig. 5). Clearly, however, the dynamics of oxidant exposure to cells differ under both conditions. When delivered as a pulse, H_2_O_2_ is depleted fairly rapidly, whereas the GOx-treated cells were exposed to a more sustained moderate concentration over the full 48-h incubation time.

### Oxidative stress index

#### Antioxidant activity of *Bucida buceras* extract

**Fig. 10:**
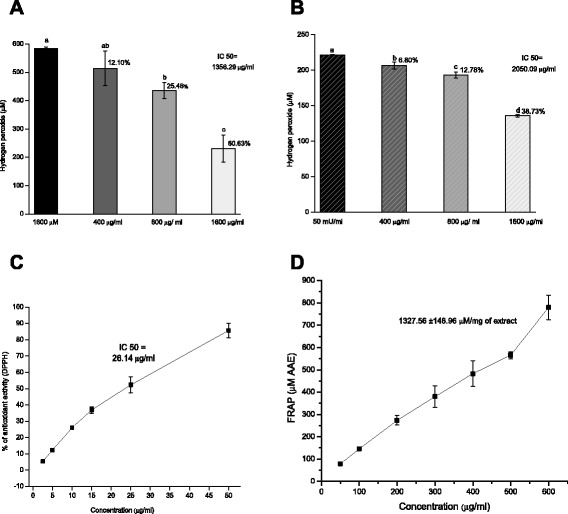
Hydrogen peroxide scavenging of ethanol *Bucida buceras* extract (400–1600 μg/ml) at 1 h in (**a**) 1600 μM H_2_O_2_ pulse y = 0.0415x - 3.2327, R^2^ = 0.99336 (IC50 = 1356.29 μg/ml) and (**b**) 50 mU/ml of GOx y = 0.0277x - 3.8591, R^2^ = 0.98872 (IC50 = 2050.09 μg/ml). (**c**) DPPH-scavenging activity of *Bucida buceras* (5 a 50 μg/ml), y = 1.6486x +6.8934, R^2^ = 0.976 IC_50_ = 26.14 μg/ml. (**d**) The ferric ion reducing antioxidant power (FRAP) in concentrations of 100 to 800 μg/ml. Values above the bars indicate % of inhibition. Each value is expressed as mean ± SD (*n* = 3)

The levels of oxidant and antioxidant capacity were simultaneously measured to assess oxidative stress and determine the antioxidant power in the control cells and cells exposed to H_2_O_2_ with or without *Bucida buceras* ethanol extract. The hydrogen peroxide scavenging activity of extract was evaluated previously. The concentration of *Bucida buceras* achieving a 50 % reduction in 1600 μM of H_2_O_2_ (pulse) and GOx 50 mU/ml (enzymatic) over 1 h is shown in Fig. 10a–b. From the results, ethanol extract showed concentration dependent activity and the H_2_O_2_ scavenging effect was higher in system of pulse delivery (IC_50_ = 1356.29 μg/ml) compared to a continuous generation (IC_50_ = 2050.09 μg/ml).

To evaluate the antioxidant activity, we started by investigating its DPPH-scavenging action. The DPPH stable free radical method is an easy, rapid, and sensitive way to survey the antioxidant activity of compounds or extracts. Figure 10c demonstrates that DPPH-scavenging potentials increased as the concentrations of *Bucida buceras* extract increased. The IC_50_ value 26.14 μg/ml was obtained through extrapolation from linear regression analysis and denoted the concentration of sample required to scavenge 50 % of DPPH radical. This result indicates the considerable antioxidant effect of *Bucida buceras* extract and can be related with phenolic compounds and carotenes present in this plant. Standards such as ascorbic acid, catechin and rutin had IC_50_ values of 7.88, 7.24 y 13.6 μg/ml respectively.

The ferric ion reducing antioxidant power (FRAP) of *Bucida buceras* extract was estimated from its ability to reduce TPTZ-Fe (III) to TPTZ-Fe (II). The intensity of blue color is a significant indicator of potential antioxidant activity. The reducing power of extract correlated well with increased concentrations (Fig. 10d). The value of reducing power was 1327.56 μM AA/mg, which suggests that the plant extract has a significant antioxidant effect. Also it may be useful for reducing free radicals, which can retard the free radical chain reaction in the propagation of the oxidation mechanism.

It is well established that the detoxification of H_2_O_2_ can be achieved through non-enzymatic (glutathione) and enzymatic ways; two different enzymatic systems, namely, catalase (CAT) and glutathione peroxidase (GPx). In this study glutathione, CAT and GPx were measured.

### Evaluation of the intracellular glutathione levels

To determine whether exposure to oxidant agents (single high dose or enzymatic generation) influenced intracellular levels of reduced glutathione, and the impact of *Bucida buceras* extract in this regard, the glutathione levels were monitored. GSH, a thiol-containing tripeptide, is the major intracellular antioxidant but it can also be the major substrate of peroxidases reducing hydroperoxides. The sulfhydryl group (SH) serves as an electron donor and reacts with ROS (H_2_O_2_, OH^.^, O_2_^.^) maintaining a reducing cellular environment.

**Fig. 11:**
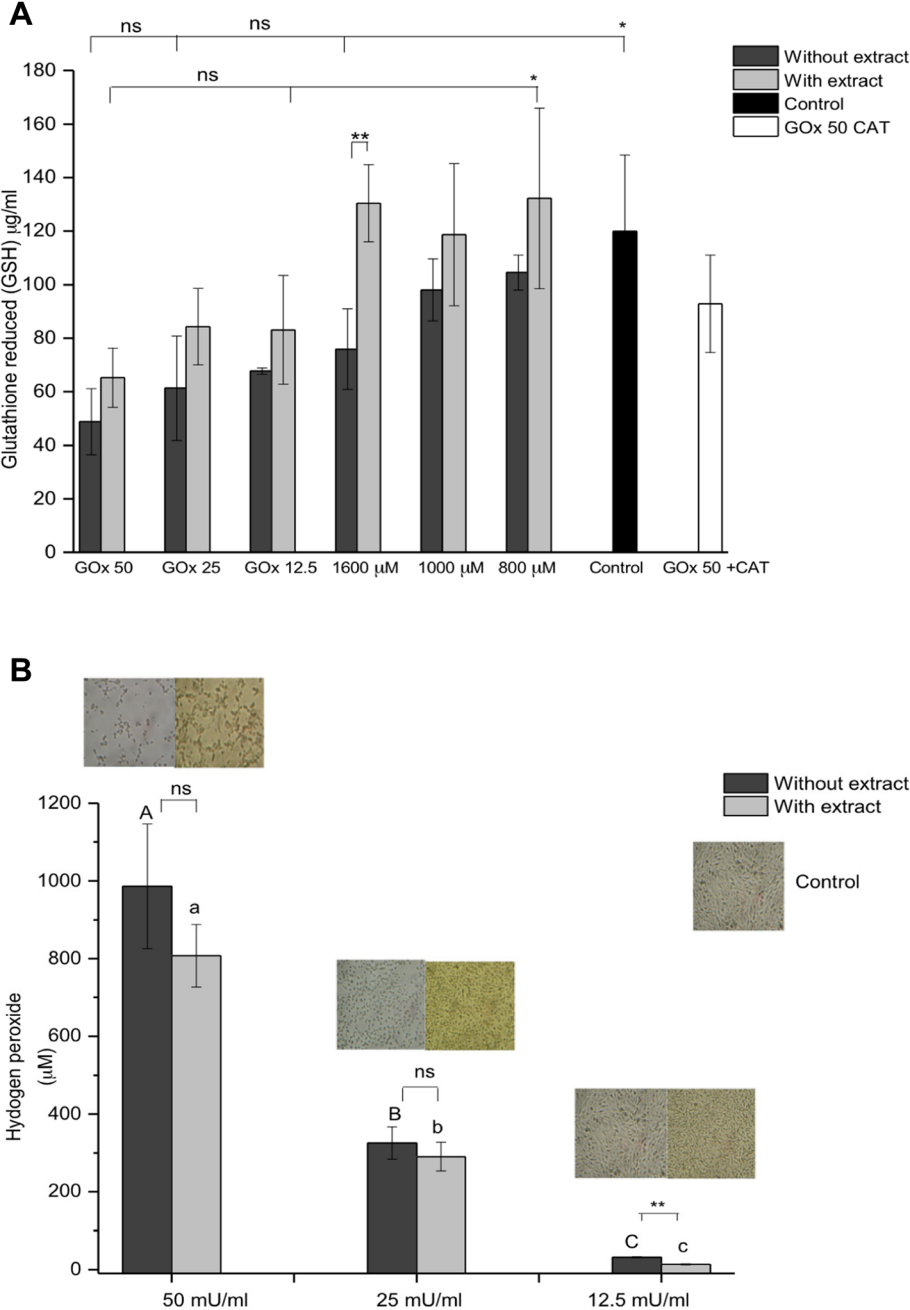
Effects of GOx and H_2_O_2_ in addition on glutathione reduced (GSH) at 24 h (**a**). ARPE-19 cells treated with 12.5, 25 and 50 mU/ml of GOx and pulse of H_2_O_2_ 800, 1000, 1600 μM and ARPE-19 cells treated with 1600 ug/ml of ethanolic extract of *Bucida buceras*, control cells (untreated with H_2_O_2_) or GOx (50 mU/ml) + 200 U/ml of catalase as CAT control; (**b**) The levels of H_2_O_2_ in GOx at 24 h in 12.5, 25 and 50 mU/ml, the medium was analyzed using FOX reagent. Values were expressed in means ± SD in μM (*n* = 3 independent experiments). *p < 0.05 significantly different from value of cells control vs. H_2_O_2_-treated **p < 0.05 significantly different from value of antioxidant treatment vs. H_2_O_2_-treated, n.s not significant differences

The results revealed that cells treated with GOx had the lowest amount of GSH, i.e., 40 to 80 μg/ml of SH content, while the cells exposed to H_2_O_2_ addition significantly increased the level (60–100 μg /ml) compared to GOx cells (Fig. 11a). As can be seen, following H_2_O_2_treatment, the glutathione levels decreased in a dose-dependent manner in ARPE-19 cells, reflecting the antioxidant protection by GSH against H_2_O_2_–induced oxidative stress. The results showed that moderate but sustained H_2_O_2_ concentrations (GOx) and pulse (1600 μM H_2_O_2_) deplete the intracellular reduced glutathione content almost entirely (*p* < 0.05). However, lower concentrations of added H_2_O_2_ (800 and 1000 μM) achieved only a minor decrease in reduced glutathione.

GSH levels were significantly higher (*p* < 0.05) in the ARPE-19 cells exposed to H_2_O_2_ with *Bucida buceras* extract and showed a 1.5 to 2-fold increased levels of GSH over the treated H_2_O_2_ cells (Fig. 11a). The increase of glutathione in cells treated with H_2_O_2_ in pulse in presence of antioxidant plant was comparable to that in the untreated control cells, indicating the possible involvement of GSH in the cytoprotection of *Bucida buceras*. Catalase inhibited the release of H_2_O_2_ from ARPE-19 cells (GOx-50 mU/ml) and the GSH increased 1.5-fold compared to cells treated with GOx (mU/ml). The lower amount of endogenous reduced glutathione indicates the increased consumption of antioxidant to counteract the elevated level of H_2_O_2._ (Fig. 11b). This behavior results mainly by the accumulation of H_2_O_2_ as a function of enzymatic concentration, whereas the delivery of H_2_O_2_ in a single pulse is not sustained.

The GSH redox cycle represents the most important H_2_O_2_ elimination pathway in the RPE cells. In our study, we found that GOx (12.5-50 mU/ml) or addition of 1600 μM H_2_O_2_ caused a dose-dependent decrease in reduced glutathione of up to 60 %, exceeding the 40 % depletion levels that render cells vulnerability to oxidative injury. *Bucida buceras* can prevent the H_2_O_2_–induced depletion of GSH, indicating that antioxidant-co-treated cells were less oxidized during oxidative stress. The recovery of GSH level may be explained by the H_2_O_2_–scavenging activity of antioxidant upon H_2_O_2_–treatment. *Bucida buceras* can increase GSH availability and as a result promote the elimination of H_2_O_2_ by increasing the catalase activity.

GSH serves as an antioxidant by scavenging ROS, which oxidize the cysteine moiety. Oxidation of GSH drives the formation of glutathione disulfide (GSSG); which can then be directly recycled to GSH through the enzyme glutathione disulfide reductase, a reaction requiring NADPH. The ratio of GSH to GSSG is often used as an indicator of intracellular redox status; more highly oxidized redox state is associated with differentiation and apoptosis. GSH can also act as a cofactor for GSH-utilizing antioxidant enzymes, such as GSH peroxidase, glutaredoxin and glutathione S-transferases.

### Evaluation of catalase (CAT) and glutathione peroxidase (GPx) activities

**Fig. 12:**
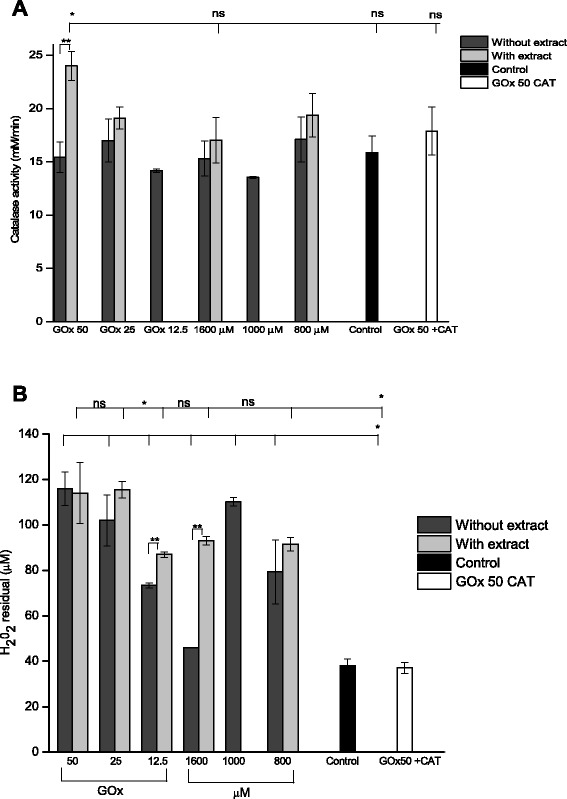
The effect of hydrogen peroxide by enzymatic generation (GOx 50, 25 and 12.5 mU/ml) and pulse (1600, 1000, 800 μM) in presence or absence of co-treatment with *Bucida buceras* ethanol extract (1600 μg/ml) on catalase activity (**a**) and GSH-peroxidase activity assay (**b**) H_2_O_2_ (initially, 200 μM in PBS was incubated for 1 h, at 37 °C with different cell lysates, control GOx (50 mU/ml + catalase 200 U/ml) or control cells (untreated with H_2_O_2_). Results are expressed as the means ± SD of three independent studies (each analyzed in triplicate). *p < 0.05 significantly different from value of cells control (untreated) vs. H_2_O_2_-treated **p < 0.05 significantly different from value of antioxidant treatment vs. H_2_O_2_-treated, n.s not significant differences

It is well established that most of the H_2_O_2_ in cells is eliminated by the intracellular antioxidant enzymes, catalase and GPx. The effect of different treatments on the activity of catalase and glutathione peroxidase is shown in Fig. 12. The CAT activity was slightly lower in the H_2_O_2_ cells than in the control cells (p > 0.05). It was found that treatment with antioxidant extract raises the activity of catalase compared to that of the untreated control and H_2_O_2_ treated . In cells treated with 50 mU/ml in co-treatment with *Bucida buceras* CAT activity increased (*p* < 0.05) (Fig. 12a). *Bucida buceras* elevates the activity of catalase indicating its preventive nature. Catalase is likely to be of particular relevance when cells are exposed to high H_2_O_2_ concentrations, due to its essentially non-saturable first-order kinetics. Catalase exerts a “filter” function that very efficiently prevents accumulation of cytosolic H_2_O_2_ levels by decomposition into H_2_O and O_2_.

GSH-peroxidase activity was evidenced by measuring the residual H_2_O_2_ during the incubation of 200 μM H_2_O_2_ with different treatments or in the presence of 500 μM GSH. We evaluated whether ARPE-19 cultures with H_2_O_2_ or GOx had different depleting effect (Fig. 12b). The untreated cells control, GOx (50 mU/ml + catalase 200 U/ml) and GSH (500 μM) had a depleting effect higher than oxidant treatments. The depletion induced by GSH is because of a passive nonenzymatic scavenging effect. In enzymatic generation the residual peroxide was diminished concentration-dependent. At the highest concentration tested (50 mU/ml), there was a decrease in the GSH-peroxidase activity as indicated by the higher H_2_O_2_ residual. In addition, the *Bucida buceras* extract had little effect on peroxidase activity. In contrast, 1600 μM of H_2_O_2_ added to the medium of ARPE-19 cells as a single pulse produces the maximum activity.

The higher CAT activity and the higher-level concentrations of glutathione appear to act as the first line of defense against H_2_O_2_ formation and oxidative damage in ARPE-19 cells co-treated with *Bucida buceras*. However GPx activity is quite low between the cells and cells with extract, suggesting that GPx has a minor role in detoxifying H_2_O_2_. An explanation for the relative importance of catalase and not of GPx in detoxifying H_2_O_2_ in ARPE-19 cells relates to the intracellular location and kinetic of these enzymes. Catalase enzyme is located in peroxisomes and its access to cytosolic is limited. However GPx, unlike catalase, is mainly present in cytosol and can be first saturated by H_2_O_2_.

**Fig. 13:**
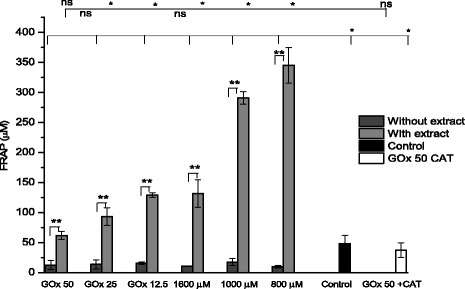
Effect of co-tretment with *Bucida buceras* (1600 μg/ml) in glucose oxidase (GOx) or H_2_O_2_ addition treatment on antioxidant capacity measured by FRAP assay. Control GOx + CAT (50 mU/ml + catalase 200 U/ml) or control cells (untreated with H_2_O_2_). *p < 0.05 significantly different from value of cells control vs. H_2_O_2_-treated **p < 0.05 significantly different from value of antioxidant treatment vs. H_2_O_2_-treated, n.s not significant differences

Antioxidant capacity by measuring the ferric reducing antioxidant power decreased after treatment with H_2_O_2_ addition or with H_2_O_2_ -generating GOx compared to culture control. As *Bucida buceras* has proposed to protect against oxidative damage, we first analyzed the effect of the antioxidant plus treatment H_2_O_2_ on power antioxidant reducing ferric (FRAP). Treatment with H_2_O_2_ or GOx for 24 h significantly decreased (*p* < 0.05) around threefold the antioxidant power in cells in the absence of extract compared to control cells (Fig. 13). Antioxidant extract treatment significantly increased (*p* < 0.05) the basal antioxidant capacity from 48.5 to 345.15 μM. Our results showed that RPE cells treated with extract exhibited a significant increase in oxidative protection in both H_2_O_2_ treatments (enzymatic and pulse). Similarly, there was an increment in antioxidant capacity of GOx-50 mU/ml plus catalase comparable to untreated cells but lower than *Bucida buceras* treatments.

### Cell protein

**Fig. 14:**
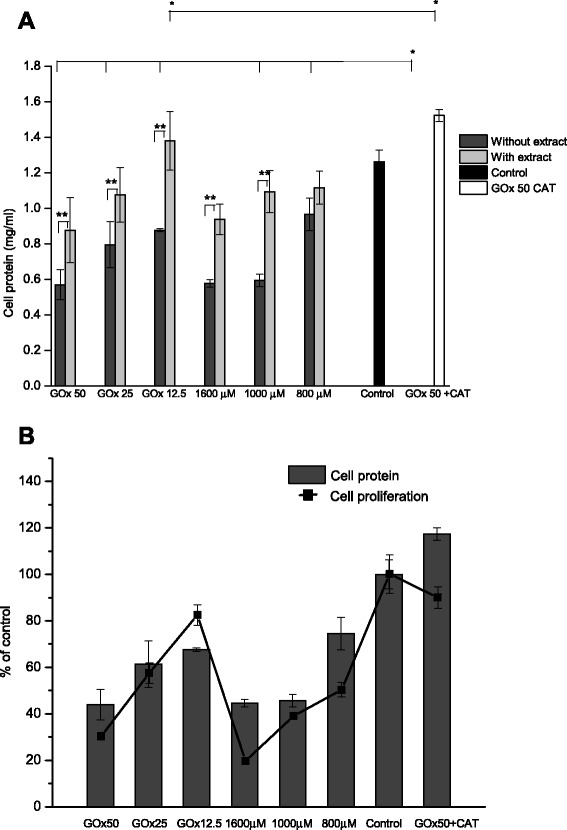
Effects of *Bucida buceras* on cell protein concentration in ARPE-19 cells H_2_O_2_-induced oxidative damage. H_2_O_2_ generated by GOx (50, 25, 12.5 mU/ml) o in addition (1600, 1000 y 800 μM) (**a**). Relationship between values for cell proliferation (line) and the protein concentration (bars) (**b**). Values are presented as means ± SD of at least 3 independent experiments. Control GOx + CAT (50 mU/ml + catalase 200 U/ml) or control cells (untreated with H_2_O_2_). *p < 0.05 significantly different from value of cells control vs. H_2_O_2_-treated **p < 0.05 significantly different from value of antioxidant treatment vs. H_2_O_2_-treated, n.s not significant differences

The significant change in cellular morphology and the increase in H_2_O_2_ of levels in ARPE-19 cells were associated with a dose-dependent decrease in protein concentrations, but antioxidant treatment prevented this decrease in cell protein (Fig. 14a). From these results it is apparent that *Bucida buceras* treatment exerted a beneficial effect on oxidative stress tolerance. The loss of protein from the cell layer compared to the untreated control cultures was related with cytotoxic action of H_2_O_2_. Because the decrease in protein concentrations could represent a cytostatic and cytotoxic effect; MTT assays were performed in control (untreated cells) and H_2_O_2_ treated cells. Whereas (100 μM of H_2_O_2_ or 5 mU/ml GOx) treatment was nontoxic at 24 h, the surviving fraction decreased approximately 50 % in 800 μM of H_2_O_2_ addition or 32.5 mU/ml GOx and 80 % in 1600 μM or 50 mU/ml GOx treated cells (Fig. 14b).

In Fig. 14b it is clearly visible that hydrogen peroxide-treated cells are losing their dividing ability in a concentration-dependent manner. The proliferating ability had not recovered after 24 h after the start of oxidative challenge. This permanent inhibition in proliferation might be attributed to the persistent presence of protein aggregates in ARPE-19 cells. 

The oxidative attack leads first to a slight protein oxidation, causing misfolding in the native protein state; if the stress persists, further oxidation aims at the hydrophobic residues that are normally buried inside proteins and these become heavily oxidized, which favors the formation of protein aggregates. Accumulation of oxidized proteins is normally degraded by the proteasome, however this process may be impaired when the rate of oxidized protein formation exceeds proteasomal capacity or when the proteasome itself has decreased its activity. The formed aggregates interact with the proteasome, inhibit its activity, and lead to a loss of functionality, e.g. proliferation.

### Effect of *Bucida buceras* extract on cell survival after exposure to H_2_O_2_

#### MTT cytotoxicity assay

**Fig. 15:**
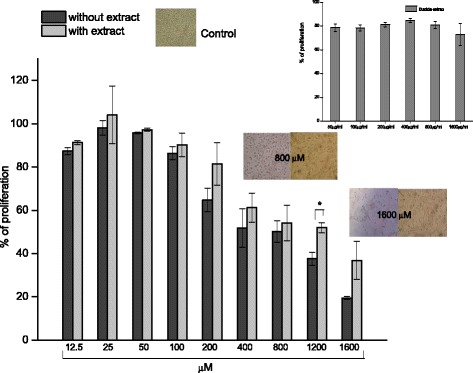
Protective effects of *Bucida buceras* on H_2_O_2_-treated ARPE-19 cells. Cells were co-treated with 1600 μg/ml in addition with various concentrations of H_2_O_2_-treatment by 24 h. Cell viability was measured by MTT assay: values are expressed as the percentage of cell survival relative to the medium control cells. Values are presented as means ± SD of 5–7 independent experiments. The insets show the low cytotoxicity of *Bucida buceras* (50 to 1600 μg/ml) on ARPE-19 cells. Cell morphology is also presented (original magnification: 200x). *p < 0.05 significantly different from value of antioxidant treatment vs. H_2_O_2_-treated alone

Cellular viability was determined through the MTT assay in ARPE-19 cells in a range of concentration 12.5 to 1600 μg/ml of extract. The present study confirmed the low toxicity of *Bucida buceras*t up to the highest concentration analyzed (Fig. 15 incept). It was further studied whether *Bucida buceras* could attenuate H_2_O_2_-stimulated ARPE-19 cell death. The Figs 5 and 9 demonstrate that exposure of ARPE-19 cells to GOx 25 or 50 mU/ml and 800–1600 μM H_2_O_2_ in addition increased the cell death.

Treatment of ARPE-19 cells with antioxidant decreased the cytotoxic effect of different oxidant treatments (Fig. 15). The co-treatment with 1600 μg/ml of extract enhanced the cell viability by ≈ 20 % compared to H_2_O_2_-treated cells alone, mainly in 1200 and 1600 μM. Exposure to 1600 μg/ml was found to exert a minor cytotoxic effect on ARPE-19 cells. Thdata indicated that *Bucida buceras* exerted a cytoprotective effect against H_2_O_2_ induced oxidative stress. This protection against H_2_O_2_-induced cell injury can be through direct H_2_O_2_ scavenging activity and its potent anti-oxidative activity.

This study demonstrates for the first time that *Bucida buceras* can prevent partially H_2_O_2_–induced cell death. It was demonstrated that treatment with extract could improve the GSH and catalase activity. This demonstrated the anti-oxidative effects of the antioxidant extract in the retinal pigmented epithelium and supports the notion that the consumption of *Bucida buceras* may provide retinal-protective effects against oxidative stress by modulating the intracellular redox status.

### Caspase-3 activity

It is well known that caspase-3 has a primordial role in triggering the cascade of events that lead to apoptosis [2, 32, 37]. Cell lysates from the experimental cultures were tested for functional caspase activity using the caspase-3-specific substrate Ac-DEVD, this peptide-PNA conjugate is noncromosphore until PNA is cleaved from the peptide by an active caspase, and consequently, increased absorbance is indicative of caspase activity.

**Fig. 16:**
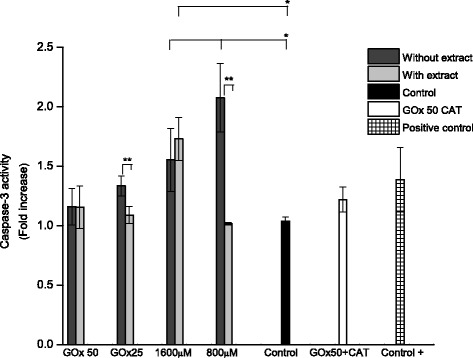
Caspase-3 activity induced by H_2_O_2_ addition (800 or 1600 μM) and GOx (25 or 50 mU/ml) in ARPE-19 cells for 24 h. Control, C^+^ cells were untreated with H_2_O, C^+^ (the specific caspase-3 control). Cells were co-treated with 1600 μg/ml of *Bucida buceras* extract. Control GOx + CAT (50 mU/ml + catalase 200 U/ml) or control cells (untreated with H_2_O_2_). Values are presented as means ± SD of at least 3 independent experiments. *p < 0.05 significantly different from value of control vs. Control GOx + CAT (50 mU/ml + catalase 200 U/ml) or control cells (untreated with H_2_O_2_) *Bucida buceras*, or H_2_O_2_-treated only; **p < 0.05 significantly different from value of antioxidant treatment vs. H_2_O_2_-treated alone

Treatment with H_2_O_2_ by addition or by enzymatic generation significantly increased the activity of caspase-3 relative to ARPE-19 control cells (Fig. 16). The control cells (untreated) showed a very minor caspase-3 activity (≈1); however, once the cells were treated with 800–1600 μM H_2_O_2,_ in addition the caspase-3 activity was increased by 1.5 and 2 fold (*p* < 0.05). Although H_2_O_2_ addition caused greater increase in the activation of caspase-3 compared to the enzymatic method. Indeed, severe oxidative stress conditions resulted in a drastic drop in the apoptosis (i.e. GOx 50 mU/ml and 1600 μM), consistent with the notion that pro-oxidant conditions can inactivate caspases by oxidative modification of key cysteine residues, thereby impairing the apoptotic program and promoting a switch to necrosis [5].

Exposure to high H_2_O_2_ concentrations (1600 μM or 50 mU/ml) exerted extensive damage leads to massive cell death. Necrotic-like cell death was reported, rather than apoptosis to high concentrations. At a moderate H_2_O_2_ cytotoxic dose (800 μM or 25 mU/ml), there was a significant increase in caspase-3 activity. Our current data further confirmed that caspase-3 might be a partial target involved in H_2_O_2_-induced apoptosis in the retinal-pigmented epithelium. On the other hand, the low correlation between caspase-3 activity and the level of cell death indicates that concentrations of H_2_O_2_ influence the mode of cell death, i.e., apoptosis vs. necrosis. The higher H_2_O_2_ concentrations induced severe toxicity, with over 90 % cell death, suggesting that under these experimental conditions necrosis was the predominant mode of cell death.

Since *Bucida buceras* treatment inhibited the cytotoxic effect of H_2_O_2_–generated oxidative stress, it was studied the direct effect on caspase-3 activity. Figure 16 shows that *Bucida buceras* exerted significant decrease on caspase-3 activity, mainly when these treatments were studied at moderate concentrations (addition system-800 μM or enzymatic-25 mU/ml)). *Bucida* extract was able to prevent H_2_O_2_-induced increase in caspase-3 activity, with reductions of 16 and 38 % in 1600 μM and 800 μM respectively. GOx 25 mU/ml + antioxidant treatment exerted similar inhibitory effect (30 % decrease) but at GOx 50 mU/ml + extract treatment did not inhibited this activity.

### Effect of *Bucida buceras* on death cell and redox status

**Fig. 17:**
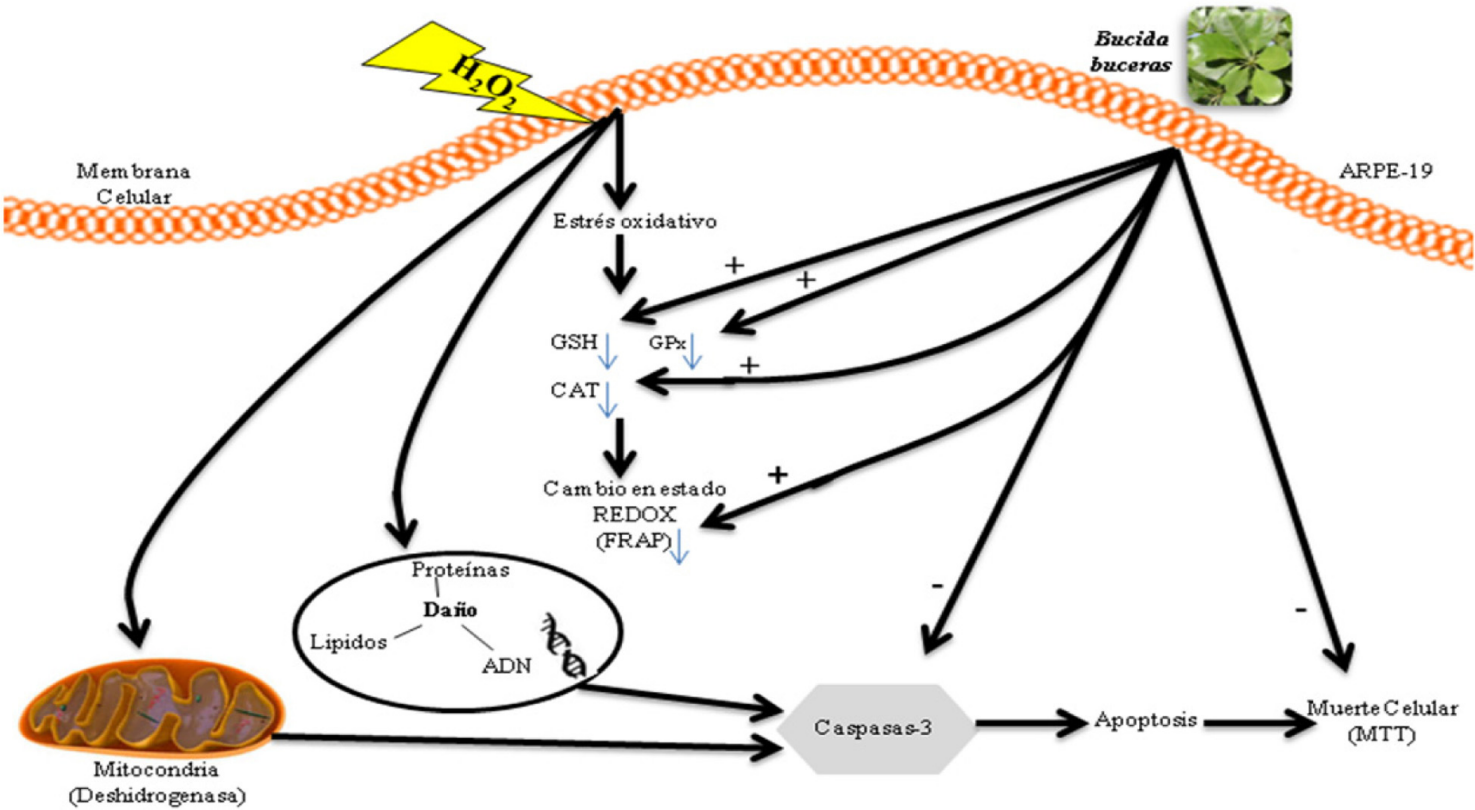
Schematic overview of the protective effects of *the Bucida buceras* on ARPE-19 cells. Co-treatment of ARPE-19 cells with antioxidant extract for 24 h protected cells against the toxicity of peroxide. H_2_O_2_ can cause oxidative stress in ARPE-19 cells as indicated by a depletion of GSH, GPx, Reducing power; *Bucida buceras* could restore GSH depletion, by enhancing the activity of glutathione peroxidase and catalase. In addition, it could also reduce H_2_O_2_-induce DNA damage by increase mitochondrial activity, and FRAP. The “+” and“-” signs indicate an increase or decrease, respectively in enzymatic activity or biochemical molecule content

Aging of the retina and RPE is associated with increased level of oxidative damage. It is generally assumed that oxidative stress also plays a role in the development or progression of age-related macular degeneration (AMD). The loss of photoreceptor function is as a consequence of oxidative damage to the RPE [1, 2]. This study employed several methods to determine whether co-treatment with extract may alter the response of ARPE-19 cells to H_2_O_2_ on cell death and redox homeostasis. The data presented showed that *Bucida buceras* extract can partially protect ARPE-19 cells against H_2_O_2_–induced cell death. The change in the intracellular redox state can increase the ability of cells to handle oxidative stress. On the other hand, treatment with antioxidant extract was able to prevent the H_2_O_2_–induced damage due to an increase in the reducing power, glutathione level, and catalase activity (Fig. 17). These findings, lead to the conclusion that regulation of cellular redox state may be the major action of *Bucida buceras*.

## Discussion

A variety of continuous RPE cell lines provide the possibility to analyze in detail their morphology, functions, and molecular and genomic properties under normal and pathological conditions, which is hardly possible *in vivo*. A major advantage of such line is that they can be subcultured over more than hundreds of passages maintaining their characteristics over a number of passages and it have longer survival times, compared to primary cultures. Another important feature is that they have a uniform cell composition, although this may be evidence that these lines have lost certain properties essential to the initial cell material. On the other hand, cell culture, as any artificial system, obviously has certain disadvantages. Unlike cells *in vivo*, cultured cells are devoid of their native 3D microenvironment.

Additional limitations on the use of RPE cell cultures arise because of the genetic instability of continuous cell lines, which results from their unstable aneuploidy chromosome constitution, and heterogeneity of short-term cultures in terms of growth rate and capacity for intrapopulation differentiation, with consequent variation in their properties between passages. Despite all these circumstances, however, cultured cells retain many specialized functions, and cell lines have become an important tool in studies on RPE. ARPE-19 cells have been widely used in studies on oxidative stress, retinal pathogenesis, and signaling pathways and also in research related to drugs and toxicity testing [12, 38–42].

Numerous studies have analyzed the response of cultured cells to H_2_O_2_ when added to the culture medium as a single o repeated bolus. Usually, the purpose of these experiments is to investigate the cellular response to “oxidative stress”, as it may occur naturally in retinal pigment epithelium that are at high risk for oxidative damage *in vivo*. Most of these studies had been conducted by adding diluted H_2_O_2_ to the cell culture medium (H_2_O_2_ bolus treatment) assuming that this at least partially mimics conditions and elicits responses that may also occur in vivo. H_2_O_2_ was utilized to generate oxidative stress due to the fact that do multiple oxidative pathways produce a common intermediate and it is involved in cellular redox signaling.

Nominal concentrations of H_2_O_2_ in cell cultures are in fact initial concentrations that are eliminated rapidly from the culture medium [5, 22, 43–47]. Exogenously added H_2_O_2_ has a short half-life cause by its rapid degradation by cellular catalase and peroxidases. Therefore amino acids present in the medium, such as methionine, cysteine and tryptophan, interact with H_2_O_2_ leading to its consumption [22, 48]. On the other hand, different supplementation of culture medium with antioxidants and precursors, as well as different contents of peroxide-consuming additives in the culture medium such as pyruvate and serum albumin is a known H_2_O_2_ scavenger [46, 49, 50]. A rapid exponential depletion of H_2_O_2_ is consistent with results reported for other cell types [46, 51] and for RPE [22]. Different supplementation of culture medium exerts variability in the cytotoxic potency of peroxides.

In our study we investigated the minimal concentration necessary to elicit 50 % of the cytotoxic effect (IC_50_). This value was calculated to be ≈ 900 μM in contrast to the found by Zhang et al. [37], who reported that ARPE-19 cells needed 24 h to develop half cytotoxicity effect at 300 μM nominal concentration but the cells were grown in serum starvation in DMEM/F-12 Su et al. [52] reported similar behavior when neuronal cells were cultured in serum-free medium for 48 h they experienced about 40 % cell death. This cell population displayed decrease in S phase and an emergence of sub-G_0_/G_1_ phase compared with those cultured in normal serum. Serum starvation successfully blocked the progression of cells from G_0_/G_1_ phase into S phase and drove cells toward apoptosis.

When H_2_O_2_ is delivered or generated outside cells, a gradient is established across the cell membrane that depends not only on the extracellular concentration of the oxidant, but also on plasma membrane permeability to H_2_O_2_ and, on the amount and availability of enzymes that decompose H_2_O_2_ [5, 44, 45]. H_2_O_2_ was found to be distributed in various subcellular compartments, i.e., the cytosol, the mitochondria, and the nucleus [5].

The low cytotoxicity of H_2_O_2_ at 4 h in ARPE-19 cells means that cytotoxicity of H_2_O_2_ takes several hours longer than the exposure to H_2_O_2_. Gulden et al. [46] revealed that the cytotoxic potency of H_2_O_2_ in C6 astroglioma cell increased about 17 fold by prolonging the incubation time. Antunes and Cadenas [44] observed that Jurkat cells needed 12 h after 1-h exposure to a steady-state concentration 25 μM H_2_O_2_ to develop maximum cytotoxicity. It has to be noted that cell lines vary in their sensitivity to H_2_O_2_. ARPE-19 cells are not very sensitive to low concentrations of H_2_O_2_ (i.e. 12.5 to 200 μM), presumably because their antioxidant defense mechanisms are sufficient to counteract the damaging effects of the low H_2_O_2_ concentrations applied. However at high H_2_O_2_ concentrations (i.e. 400–1600 μM), the antioxidant defense mechanism in the ARPE-19 cell line appears to be insufficient to cope with such high oxidant insult, resulting in a high percentage of cell death.

The retinal pigmented epithelium is armed with a robust antioxidant system because of the high ambient oxygen tension required to maintain its high metabolism that is necessary to maintain the health and function of the overlying photoreceptors, and the unique, constant exposure to photo-oxidative stress [53]. Despite a potent antioxidant system, the RPE cell became progressively dysfunctional and finally died by apoptosis. Part of this deterioration has been attributed to inadequately neutralized oxidative stress. Oxidative damaged in biomolecules have been identified in the RPE from AMD samples [53, 54].

GSH had an essential role in preserving nuclear function from deleterious oxidative modifications. A minimal GSH concentration is essential to protect the nucleus against oxidative damage and the maintaining of nuclear function during mild H_2_O_2_ treatment is the key parameter of cell survival. When GSH concentration is high (>1 mM), all cell functions are preserved from oxidative inhibition. As a result, enzymes of the detoxification machinery are induced and intracellular H_2_O_2_ is rapidly degraded. At a lower concentration (≈0.1 mM), GSH loses its cytosolic protective effect but it remains efficient for the protection of the nucleus against oxidative damage. Despite the transcriptional induction of stress-responsive genes, the detoxification machinery remains at a low level, because of the inhibition of translation. Therefore, is a slowly degraded and oxidized protein accumulating in the cytosol. But upon stress release, the functional nucleus can support growth resumption. When GSH concentration drops under 0.03 mM, both nuclear and cytosolic components become highly sensitive to oxidative damage, leading to cell death [55].

The protective effect of *Bucida buceras* in the cell viability of ARPE-19 is comparable to results reported in human colorectal cancer cells. Where cells exposed to 0.5 mM H_2_O_2_ treatment with 8 mM GSH increased cell viability by ≈ 15 % and reduced oxidative microsatellite mutations by ≈ 75 %. However N-acetylcysteine (NAC) added at 10 mM did not further affect cell viability that had already been reduced by 0.5 mM H_2_O_2_ although NAC suppressed oxidative microsatellite mutations [56].

Progression of death stimuli to apoptosis and necrosis depended on the mitochondrial-mediated damage and on ATP levels. The presence of ATP is essential for the activation of apoptosis protease and subsequent activation of caspases that induce apoptosis. The data obtained from this work indicate that moderate oxidative stress induced by H_2_O_2_ is capable of inducing cell death by apoptosis dependent of caspase-3 in ARPE-19. Yu et al. [57] found that cell injury with 200 μM H_2_O_2,_ in human lens epithelial B3 (HLE B3) is mediated primarily by caspase-associated apoptosis. HLE B3 cells were grown in serum deprivation MEM before exposure to a bolus of H_2_O_2_. While Zhang et al. [37] found that the aqueous extract of the root of *Salvia miltiorrhiza* (Salvianolic acid A) inhibited H_2_O_2_-induced caspase-3 cleavage in RPE cells. The cytoprotective effect of Sal A on RPE cells was also due to the activation of the transcription factor Nrf2 and its dependent antioxidant-responsive element (ARE)- genes.

In the RPE, oxidative stress is a powerful risk factor for age-related macular degeneration (AMD) and retinopathy diseases. The susceptibility of RPE cells to oxidative stress progressively increases with age, and cumulative oxidative damage contributes to RPE dysfunction and apoptosis [58]. Thurman et al. [59] found that H_2_O_2_ depleted nuclear factor erythroid-derived 2-related factors 2 (Nrf2). Nrf2 regulates the inducible expression of antioxidant and cytoprotective enzymes via the cis-acting antioxidant response element. The *Bucida buceras* extract may affect genomic expression, which in turn regulates the expression of certain cytoprotective enzymes (i.e., catalase or GPx). We speculate therefore that long-term increment in antioxidant status will probably reduce RPE oxidative damage in the human eye and may delay onset of age-related visual impairment.

## Conclusion

These findings suggest that *Bucida buceras* could protect RPE against ocular pathogenesis associated with oxidative stress induced by H_2_O_2_-delivered by addition and enzymatic generation. The change in the intracellular redox state can increase the ability of cells to handle oxidative stress. On the other hand, treatment with antioxidant extract was able to prevent the H_2_O_2_–induced damage due to an increase in the reducing power, glutathione level, and catalase activity. The regulation of cellular redox state may be the major action of *Bucida buceras*.

## References

[1] Miranda M, Arnal E, Ahuja A, Alvarez R, López R, Ekstrom P, Romero F. Antioxidants rescue photoreceptors in rd1 mice: Relationship with thiol metabolism. Free Radic. Biol. Med. 2010; 48:216–22. 10.1016/j.freeradbiomed.2009.10.042 19854264

[2] Yu CC, Nandrot EF, Dun Y, Finnemann SC. Dietary antioxidants prevent age-related retinal pigment epithelium actin damage and blindness in mice lacking αvβ5 integrin. Free Radical Biology and Medicine 2012;52:660–70. 10.1016/j.freeradbiomed.2011.11.021 PMC3267844 22178979

[3] Bian Q, Gao S, Zhou J, Qin J, Taylor A, Johnso EJ, et al. Lutein and zeaxanthin supplementation reduces photooxidative damage and modulates the expression of inflammation-related genes in retinal pigment epithelial cells. Free Radical Biology and Medicine, 2012;53:1298–307. 10.1016/j.freeradbiomed.2012.06.024 PMC3744865 22732187

[4] Lewis LLM, Iloki ASB, Fernández AD, Gil SAA, Lara ECL, Rubio PJL. Oxidative stress implications for the pathogenesis of ocular pathology. International Journal of Development Research 2015;5(2)3275–288, February.

[5] Panieri E, Gogvadze V, Norberg E, Venkatesh R, Orrenius S, Zhivotovky B. Reactive oxygen species generated in different compartments induce cell death, survival, or senescence. Free Radical Biology and Medicine, 2013;57:176–87. 10.1016/j.freeradbiomed.2012.12.024 23295411

[6] Bienert GP, Schjoerring JK, Jahn TP. Membrane transport of hydrogen peroxide. Biochim. Biophys. Acta, 2006;1758:994–1003. 10.1016/j.bbamem.2006.02.015 16566894

[7] Mittler R, Vanderauwera S, Suzuki N, Miller G, Tognetti VB, Vandepoele K et al. ROS signaling: the new wave? Trends Plant Sci. 2011;16:300–309. 10.1016/j.tplants.2011.03.007 21482172

[8] Rhee SG, Bae YS, Lee SR, Kwon J. Hydrogen peroxide: a key messenger that modulates protein phosphorylation through cysteine oxidation. Sci. STKE 2000, pe1. 10.1126/stke.2000.53.pe1 11752613

[9] Finkel T. Signal transduction by reactive oxygen species. J. Cell Biol. 2011, 194:7–15. 10.1083/jcb.201102095 PMC3135394 21746850

[10] Zhu Y, Park SH, Ozden O, Kim HS, Jiang H, Vassilopoulos A et al. Exploring the electrostatic repulsion model in the role of Sirt3 in directing MnSOD acetylation status and enzymatic activity. Free Radic. Biol. Med. 2012;53:828–33. 10.1016/j.freeradbiomed.2012.06.020 PMC3418453 22732184

[11] Eno CO, Zhao G, Venkatanarayan A, Wang B, Flores ER, Li C. Noxa couples lysosomal membrane permeabilization and apoptosis during oxidative stress. Free radical Biology and Medicine. 2013;65:26–37. 10.1016/j.freeradbiomed.2013.05.051 PMC3816129 23770082

[12] Kim YN, Jung HY, Eum WS, Kim DW, Shin MJ, Ahn EH et al. Neuroprotective effects of PEP-1-carbonyl reductase 1 against oxidative stress induced ischemia neuronal cell damage. Free radical Biology & Medicine, 2014;69:181–196. 10.1016/j.freeradbiomed.2014.01.006 24440593

[13] Pickering AM, Linder RA, Zhang H, Forman HJ, Davies KJ. Nrf2-dependent induction of proteasome and Pa28alphabeta regulator are required for adaptation to oxidative stress. J. Biol. Chem. 2012;287:10021–31. 10.1074/jbc.M111.277145 PMC3323025 22308036

[14] Mannermaa E. In vitro model of retinal pigment epithelium for use in drug delivery studies [Dissertations in Health Sciences], 2010, University of Eastern Finland, Kuopio, Finland.

[15] Ahmado AAJ, Carr AA, Vugler M, Semo C, Gias JM, Lawrence LL et al. Induction of differentiation by pyruvate and DMEM in the human retinal pigment epithelium cell line ARPE-19. Investig. Ophthalmol. Vis. Sci. 2011;52:7148–59. 10.1167/iovs.10-6374 21743014

[16] Boulton ME. Studying melanin and lipofuscin in RPE cell culture models. Experimental Eye Research. 2014;126:61–67. 10.1016/j.exer.2014.01.016 PMC4143628 25152361

[17] Burke JM, Hjelmeland LM. Mosaicism of the retinal pigment epithelium: seeing the small picture. Mol Interv. 2005;5:241–249. 10.1124/mi.5.4.7 16123538

[18] Strauss O. The Retinal Pigment Epithelium in Visual Function. Physiol. Rev. 2005;85:845–854. 10.1152/physrev.00021.2004 15987797

[19] Tanito M, Nishiyama A, Tanaka T, Masutani H, Nakamura H, Yodoi J, et al. Change of redox status and modulation by thiol replenishment in retinal photo-oxidative damage. Invest Ophthalmol Visual Sci. 2002;43:2392–400. 12091442

[20] Wu J, Seregard S, Algvere PV. Photochemical damage of the retina. Surv Ophthalmol. 2006;51:461–81. 10.1016/j.survophthal.2006.06.009 16950247

[21] Korytowski W, Pilas B, Sarna T, Kalyanaraman B. Ptotoinduced generation of hydrogen peroxide and hydroxyl radicals in melanins. Photochem Photobiol. 1987;45:185–90. 10.1111/j.1751-1097.1987.tb05362.x 3031709

[22] Kaczara P, Sarna T, Burke JM. Dynmics of H_2_O_2_ availability to ARPE-19 culture in models of oxidative stress. Free Radic Biol Med. 2010;48:1064–70. 10.1016/j.freeradbiomed.2010.01.022 PMC2839027 20100568

[23] Kim M-H, Jin C, Yang J-W, Chung S-M, Kwag Yoo J-S. Hydrogen Peroxide-Induced Cell Death in a Human Retinal Pigment Epithelial Cell Line, ARPE-19. Korean J Ophthalmol. 2003;17(1):19–28. 10.3341/kjo.2003.17.1.19 12882504

[24] Hayashi K, Yuka N, Bastow KF, Gordon C, Hiroshi Nozaki B, Kuo-Hsiung L. Antitumor Agents. Part 212: Bucidarasins A–C, Three New Cytotoxic Clerodane Diterpenes from Bucida buceras. Bioorg Med Chem Lett. 2002;12:345–8. 10.1016/s0960-894x(01)00742-9 11814793

[25] Hayashi K, Yuka N, Bastow KF, Cragg G, Hiroshi Nozaki B, Kuo-Hsiung L. Antitumor Agents. Part 221: Antitumor Agents. 221.1 Buceracidins A and B, Two New Flavanones from *Bucida buceras*. J Nat Prod. 2003;66:125–7. 10.1021/np0203483 12542360

[26] Adonizio A, Kong K-F, Mathee K. Inhibition of Quorum Sensing-Controlled Virulence Factor Production in Pseudomonas aeruginosa by South Florida Plant Extracts. Antimicro Agents Chemotherapy. 2008;52:198–203. 10.1128/AAC.00612-07 PMC2223872 17938186

[27] Mahlo SM, McGaw LJ, Eloff JN. Antifungal activity of leaf extracts from South African trees against plant pathogens. Crop Prot. 2010;29(12):1529–33.

[28] Mahlo SM, Chauke HR, McGaw LJ, Eloff JN. Antioxidant and antifungal activity of selected plant species used in traditional medicine. J Med Plants Res. 2013;7(33):2444–50.

[29] Gil L, Martinez G, González I, Tarinas A, Alvarez A, Giuliani R, et al. Contribution to characterization of oxidative stress in HIV/AIDS patients. Pharmacol Res. 2003;47(3):217–24. 10.1016/s1043-6618(02)00320-1 12591017

[30] Mosmann T. Rapid colorimetric assay for cellular growth and survival: application to proliferation and cytotoxicity assays. J Immunol Methods. 1983;65:55–63. 10.1016/0022-1759(83)90303-4 6606682

[31] Bradford MM. A rapid and sensitive method for the quantification of microgram quantities of protein utilizing the principle of protein-dye binding. Anal Biochem. 1976;72:248–54. 10.1016/0003-2697(76)90527-3 942051

[32] Bai YP, Hu CP, Yuan Q, Peng J, Shi R, Shi RZ, et al. Role of VPO1, a newly identified heme-containing peroxidase, in ox-LDL induced endothelial cell apoptosis. Free Radic Biol Med. 2011;51:1492–500. 10.1016/j.freeradbiomed.2011.07.004 PMC3570029 21820048

[33] Benzie FF, Strain JJ. “Ferric reducing ability of plasma (FRAP) as a measure of antioxidant power: The FRAP assay”. *Analytical Biochemistry*. 1996;239(1):70–76. 10.1006/abio.1996.0292 8660627

[34] Iloki S, Lewis L, Rivera G, Gil A, Acosta A, Meza C, et al. Effect of maturity and harvest season on antioxidant activity, phenolic compounds and ascorbic acid of *Morinda citrifolia L*. (noni) grown in Mexico. Afr J Biotechnol. 2013;12(29):4630–9.

[35] Brand-William W, Cuvelier ME, Berset C. Use of a free radical method to evaluate antiozidant activity. Lebensmittel-Wissenchaft and Technol. 1995;28:25–30.

[36] Lewis LLM, Iloki ASB, Rivera CEG, Gil SAA, Acosta SAL, Meza CCY (2014). Nutritional and Phenolic composition of *Morinda citrifolia* L. (Noni) fruit at different ripeness stages and seasonal patterns harvested in Nayarit, Mexico. Int J Nutri Food Sci.

[37] Zhang H, Liu YY, Jiang Q, Li KR, Zhao YX, Cao C, et al. Salvianolic acid A protects RPE cells against oxidative stress through activation of Nrf2/HO-1 signaling. Free Radic Biol Med. 2014;69:219–28. 10.1016/j.freeradbiomed.2014.01.025 24486344

[38] Freshney RI. Culture of Animal Cells: A Manual of Basic Technique. 5th ed. Hoboken, NJ, USA: John Wiley & Sons; 2005.

[39] Cai H, del Priore LV. Gene expression profile of cultured adult compared to immortalized human retinal pigment epithelium. Mol Vis. 2006;12:1–14. 16446697

[40] Fragoso MA, Patel AK, Nakamura REI, Yi H, Sur– apaneni K, Hackam AS. The Wnt /*β*-catenin pathway cross- talks with STAT3 signaling to regulate survival of retinal pigment epithelium cells. PLoS One. 2012;7:10. Article ID e46892. 10.1371/journal.pone.0046892 PMC3464242 23056515

[41] Dithmer M, Fuchs S, Shi Y, Schmidt H, Richert E, Roide J, et al. Fucoidan reduces secretion and expression of vascular endothelial growth factor in the retinal pigment epithelium and reduces angiogenesis in vitro. Plos One. 2014;9:2. 10.1371/journal.pone.0089150 PMC3928431 24558482

[42] Kuznetsova, A. V., Kurinov, A. M. and Aleksandrova M.A. Cell Models to Study Regulation of Cell Transformation in Pathologies of Retinal Pigment Epithelium. Journal of Ophthalmology Volume 2014, Article ID 801787 10.1155/2014/801787 PMC4142280 25177495

[43] Dringen R, Hammprecht B. Involvement of glutathione peroxidase and catalase in the disposal exogenous hydrogen peroxide by cultured astroglial cells. Brain Res. 1997;759:67–75. 10.1016/s0006-8993(97)00233-3 9219864

[44] Antunes F, Cadenas E. Estimation of gradient across biomembranes. FEBS Lett. 2000;475:121–6. 10.1016/s0014-5793(00)01638-0 10858501

[45] Makino N, Mise T, Sagara J. Kinetics of hydrogen peroxide elimination by astrocytes and C6 glioma cells: analysis based on a mathematical model. Biochim Biophys Acta. 2008;1780:927–36. 10.1016/j.bbagen.2008.03.010 18402782

[46] Gulden M, Jess A, Kammann J, Masr E, Seibert H. Cytotoxic potency of in cell cultures: Impact of cell concentration and exposure time. Free Radic Biol Med. 2010;49:1298–305. 10.1016/j.freeradbiomed.2010.07.015 20673847

[47] Qenaei AA, Yiakouvaki A, Reelfs O, Santambrogio P, Levi S, Hall ND (2014). Role of intracellular labile iron, ferritin, and antioxidant defense in resistance of chronically adapted Jurkat T cell to hydrogen peroxide. Free Radic Biol Med.

[48] Kim YH, Berry AH, Spencer DS, Stites WE. Comparing the effect on protein stability of methionine oxidation versus mutagenesis: steps toward engineering oxidative resistance in proteins. Protein Eng. 2001;14:343–7. 10.1093/protein/14.5.343 11438757

[49] Andrae U, Singh J, Ziegler-Skylakakis K. Pyruvate and related α-ketoacids protect mammalian cells in culture against hydrogen peroxide-induced cytotoxicity. Toxicol Lett. 1985;28:93–8. 10.1016/0378-4274(85)90015-3 4071565

[50] Carballal S, Radi R, Kirk MC, Barnea S, Freeman BA, Alvarez B. Sulfenic acid formation in human serum albumin by hydrogen peroxide and peroxinitrite. Biochemistry. 2003;42:9906–14. 10.1021/bi027434m 12924939

[51] Sobotta MC, Barata AG, Schmidt U, Mueller S, Millonig G, Dick TP. Exposing cells H_2_O_2_: A quantitative comparison between continuous low-dose and one-time high-dose treatments. Free Radic Biol Med. 2013;60:325–35. 10.1016/j.freeradbiomed.2013.02.017 23485584

[52] Su JD, Yen JH, Li S, Weng CY, Lin MH (2012). 3, 4Didemethhylnobiletin induces phase II detoxification gene expression and modulates P13K/Akt signaling in PC12 cells. Free Radic Biol Med.

[53] Cano M, Wang L, Wan J, Bradley PB, Ebrahimi K, Qian J, et al. Oxidative stress induces mitochondrial dysfunction and a protective unfolded protein response in RPE cells. Free Radic Biol Med. 2014;69:1–14. 10.1016/j.freeradbiomed.2014.01.004 PMC3960355 24434119

[54] Del Priore LV, Kuo YH, Tezel TH. Age-related changes in human RPE cell density and apoptosis proportion in situ. Invest Ophthalmol Visual Sci. 2002;43:3312–8. 12356840

[55] Hatem E, Berthonaud V, Dardalhon M, Lagniel G, Cornu PB, Huang ME, et al. chedin, S. Glutathione is essential to preserve nuclear function and cellsurvival under oxidative stress. Free Radic Biol Med. 2014;67:103–14. 10.1016/j.freeradbiomed.2013.10.807 24145121

[56] Li IC, Chiu CY, Wu CL, Chi JY, Jian SR, Wang SW, et al. A dual-fluorescent reporter facilities identification of thiol compounds that suppress microsatellite instability induced by oxidative stress. Free Radic Biol Med. 2014;69:86–95. 10.1016/j.freeradbiomed.2013.12.019 24412704

[57] Yu Y, Xing K, Badama R, Kuszynski CA, Wu H, Lou MF (2013). Overexpression of thioredoxin-binding protein 2 increases oxidation sensitivity and apoptosis in human lens epithelial cells. Free Radic Biol Med.

[58] Wang L, Kondo N, Cano M, Ebrahimi K, Yoshida T, Barnett BP, et al. Nrf2 signaling modulates cigarette smoke-induced complement activation in retinal pigmented epithelial cells. Free Radic Biol Med. 2014;70:155–66. 10.1016/j.freeradbiomed.2014.01.015 PMC4006310 24440594

[59] Thurman JM, Renner B, Kunchithapautham K, Ferreira VP, Pangburn MK, Ablonczy Z, et al. Oxidative stress renders retinal pigment epithelial cells susceptible to complement-mediated injury. J Biol Chem. 2009;284:16939–47. 10.1074/jbc.M808166200 PMC2719331 19386604

